# Integrating multimodal cancer data using deep latent variable path modelling

**DOI:** 10.1038/s42256-025-01052-4

**Published:** 2025-07-22

**Authors:** Alex Ing, Alvaro Andrades, Marco Raffaele Cosenza, Jan O. Korbel

**Affiliations:** 1 Genome Biology Unit, European Molecular Biology Laboratory, Heidelberg, Germany; 2 Bridging Research Division on Mechanisms of Genomic Variation and Data Science, German Cancer Research Center (DKFZ), Heidelberg, Germany

**Keywords:** Breast cancer, Breast cancer, Data integration, Machine learning, Computer science

## Abstract

Cancers are commonly characterized by a complex pathology encompassing genetic, microscopic and macroscopic features, which can be probed individually using imaging and omics technologies. Integrating these data to obtain a full understanding of pathology remains challenging. We introduce a method called deep latent variable path modelling, which combines the representational power of deep learning with the capacity of path modelling to identify relationships between interacting elements in a complex system. To evaluate the capabilities of deep latent variable path modelling, we initially trained a model to map dependencies between single-nucleotide variant, methylation profiles, microRNA sequencing, RNA sequencing and histological data using breast cancer data from The Cancer Genome Atlas. This method exhibited superior performance in mapping associations between data types compared with classical path modelling. We additionally performed successful applications of the model to stratify single-cell data, identify synthetic lethal interactions using CRISPR–Cas9 screens derived from cell lines and detect histologic–transcriptional associations using spatial transcriptomic data. Results from each of these data types can then be understood with reference to the same holistic model of illness.

## Abstract (web summary)

Cancers have complex genetic, microscopic and macroscopic features analysed using imaging and omics technologies, but integrating these data is challenging. A framework combining deep learning and path modelling to integrate imaging and omics data is presented, producing a unified model of disease.

## Main

Many common illnesses such as cancer, cardiovascular diseases and neurological disorders result from complex pathologies that possess genetic, microscopic and macroscopic components^1–4^. Over recent decades, the invention and widespread use of diverse omics and imaging technologies has provided important insights into the mechanisms that underpin these diseases^5^. However, analysed individually, these technologies may illuminate only a single aspect of pathology. A comprehensive understanding of complex disease necessitates the integration of these disparate data types^6^. There is a pressing need for new methods designed for this purpose.

Cancers are characterized by intricate pathological mechanisms. Cellular functions are dictated via multiple layers of biological information and processing. In cancer, this information is corrupted, and normal processes are subverted, giving cancer cells the ability to survive, proliferate and metastasize^1^. Recent studies have revealed a diversity of somatic mutation classes, widespread epigenetic changes and substantial alterations in gene expression, all of which exhibit high heterogeneity across tumours, even within the same tissue^7^. Despite its molecular genesis, cancer is still primarily diagnosed and understood clinically through histological imaging; this process involves extracting, sectioning, staining and imaging tumour biopsies to identify aberrations in tissue microstructure linked to specific clinical phenotypes^8^. Efficient, integrative approaches that systematically combine multiomic and imaging modalities could lead to deeper insights into cancer biology.

Deep learning methods excel at processing unstructured data, such as imaging, by identifying complex patterns without explicit programming^9^. In recent years, numerous hypothesis-driven studies have been conducted with a focus on predicting the presence of genetic characteristics of cancer, such as driver mutations and clinical status, using histological data^10,11^. Deep learning methods have also proved highly effective in modelling the complex interdependencies between genes in multiomic datasets^12,13^. Other research efforts have aimed to integrate histological and genetic data to predict clinically important outcomes, including patient survival times^14–18^ and drug response profiles^19^. Despite these advancements, exploratory research that seeks to map the complex interactions between various layers of genetic information and histological data is still in its infancy, presenting a largely uncharted frontier in oncology. All-encompassing methods are required to comprehensively map the causal and statistical dependencies between different data types relevant in cancer biology.

Path modelling (also called structural equation modelling) is a powerful and widely used class of techniques used primarily in epidemiology^20^, social sciences^21^ and econometrics^22^. Intuitively, a path model can be thought of as a map, specifying connections between different data types. Path-modelling methods are ideally suited for multimodal data integration in biology as they allow the simultaneous estimation of multiple relationships, enabling the detailed examination of both direct and indirect effects between different data modalities. Furthermore, the visual representation of results in path diagrams aids in intuitively communicating complex inter-relations. Path-modelling methods are self-supervised in the sense that they are not trained for a specific task, but rather to learn the underlying structure of a multimodal dataset. Once the model is trained, it can be applied to a variety of downstream tasks, such as prediction, classification and even causal inference^23,24^. Despite these strengths, path-modelling methods currently struggle with representing and capturing the complexity of unstructured data types, such as images. Consequently, they share similar constraints with classical techniques used for classification and regression, which are inadequate for the evaluation of unstructured data, and the handling of complex, nonlinear patterns frequently encountered in biological research^12,13^.

In this study, we introduce a deep-learning-based method for path modelling called deep latent variable path modelling (DLVPM). This method combines the representational power of deep learning, with the ability of path modelling to map complex dependencies between data types. In the cancer context, this allows us to model the genetic and epigenetic interactions with gene expression, which, in turn, result in the microscopically visible aberrations in tissue structure that are a characteristic of cancer. A crucial strength of the method is its modular nature, which allows submodels trained for each individual modality to be characterized further on new datasets.

We trained a full DLVPM path model on The Cancer Genome Atlas (TCGA) breast cancer dataset^25^, one of the most comprehensive and well-annotated datasets combining imaging and multiomics data modalities. However, before initiating full path modelling, we pretrained a histological model, again using DLVPM, which was benchmarked against other state-of-the-art methods and histological models. This model served as a foundation for the integration of histological data into the full path-modelling framework. The DLVPM method proved superior to classical path modelling in identifying inter-relations among genetic, epigenetic and histological data. In secondary analyses, we used this model to identify hundreds of genetic loci showing an individually significant association with histology. The molecular subcomponent of the full DLVPM model, initially trained on the TCGA patient data, was then successfully replicated on independent patient and cell-line data, and used to explore the differential sensitivity of breast cancer cell lines to CRISPR–Cas9 knockouts, revealing significant associations between the model and many gene dependencies. Spatial transcriptomic data were then used to further characterize these genes in the context of the DLVPM model. This approach offers a holistic view of cancer, illustrating the power of DLVPM as a singular, comprehensive method for multilayered data integration.

## Results

### DLVPM

DLVPM is a framework that unites the flexibility of deep neural networks^9^ with the interpretability and structure of path modelling^23,26,27^. By leveraging powerful representations learned through neural architectures, DLVPM extends classical path modelling beyond linear relationships and latent variables to rich, nonlinear embeddings.

**Fig. 1:**
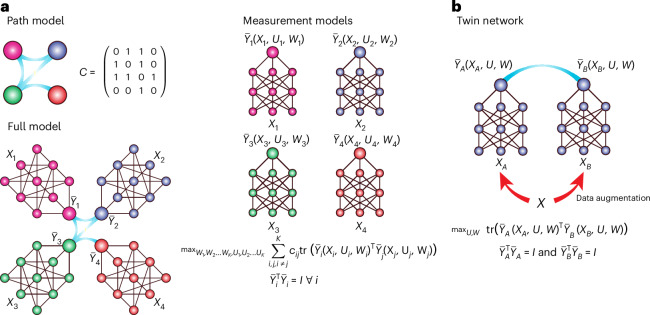
Schematic illustrating the DLVPM algorithm. **a**, Constituent parts of a DLVPM model. The path model defines the data types that are connected to one another. The measurement models for each data type are used to construct the DLVs that are optimized to be strongly correlated between data types. The overall model combines both path model and measurement models. This image represents DLVPM in a situation where four data types are available. **b**, Use of DLVPM in a Siamese/twin network configuration. Here augmented versions of the same input are fed to a network, and the network is trained to learn DLVs that are invariant to these augmentations.

Path-modelling/structural-equation-modelling methods are a family of procedures used for mapping dependencies between different data types^23,26,27^. These methods are able to model arbitrarily many data types simultaneously, providing a holistic view of a system of interacting elements. Path-modelling analyses begin with the user specifying the path model itself. This model encodes hypotheses about the relationships between data types included in an analysis. These models are usually represented visually as a network graph (Fig. 1a), and mathematically as an adjacency matrix.

$$C=\left(\begin{array}{cc}\begin{array}{cc}{c}_{11} & {c}_{12}\\ {c}_{21} & {c}_{22}\end{array} & \begin{array}{cc}\cdots & {c}_{1K}\\ \cdot & {c}_{2K}\end{array}\\ \begin{array}{cc}\vdots & \cdot \\ {c}_{K1} & {c}_{K2}\end{array} & \begin{array}{cc}\cdot & \vdots \\ \cdots & {c}_{KK}\end{array}\end{array}\right)c_{ij}\in\{0,1\}\;\forall i,j$$

The adjacency matrix is a square matrix, where elements *c*_*ij*_ represent connections between data types *i* and *j* and *K* is the total number of data types under analysis. Each element in the matrix indicates the presence (value of one) or absence (zero value) of a direct influence from one data type to another.

In classical path modelling, techniques like partial least squares path modelling (PLS-PM) are used to derive latent variables that exhibit optimal correlation among datasets linked by the path model. However, such techniques are limited to modelling linear effects^23^.

Deep neural networks excel in their ability to model nonlinear effects, and to process structured and unstructured data. Most neural networks can be written in the general form $\bar{Y}(X,U)$, where $\bar{Y}$ is the network output, *X* is some data input and *U* is the set of network parameters (including weights, biases and other network parameters).

In DLVPM, we define a collection of submodels, one for each data type, indexed here by the subscript *i*:

$${\bar{Y}}_{i}({X}_{i},\,{U}_{i},{W}_{i}),$$

where ${\bar{Y}}_{i}$ is the network output, a set of deep latent variables (DLVs), *U*_*i*_ is the set of parameters up to the penultimate network layer and *W*_*i*_ corresponds to the network weights on the last layer of the neural network. This weight is displayed separately as it represents a linear projection and is critical to the way DLVPM is trained. These submodels are called measurement models^23^.

The DLVPM algorithm is then trained to construct DLVs from each measurement model, which are optimized to be maximally associated with DLVs from other measurement models, connected by the path model. These optimization criteria can be written as

$$\max_{{W}_{1},{W}_{2},\ldots, {W}_{K},{U}_{1},{U}_{2},\ldots, {U}_{K}}\mathop{\sum }\limits_{i,\,j,i\ne j}^{K}{c}_{ij}{\rm{tr}}({\bar{Y}}_{i}{({X}_{i},{U}_{i},{W}_{i})}^{{\rm{T}}}{\bar{Y}}_{j}({X}_{j},\,{U}_{j},{W}_{j})),$$

where *c*_*ij*_ represents the association matrix input from data type *i* to data type *j*, and tr denotes the matrix trace. DLVs derived from each data type are constrained to be orthogonal to one another:

$${{\bar{Y}}_{i}}^{{\rm{T}}}{\bar{Y}}_{i}=I\;\forall i,$$

where *I* is the identity matrix. These DLVs are then optimized to be strongly correlated across data types connected by the path model, while maintaining orthogonality within each data type, thereby capturing the essence of each data type’s contribution to the system and minimizing information redundancy within the model. Following model training, the DLVPM algorithm results in a set of orthogonal path models representing associations between DLVs constructed from each data type. In deep learning parlance, these DLVs can be considered to represent a joint embedding.

DLVPM’s training process is both iterative and end to end, enabling the model to learn directly from the raw data to the final output without the requirement for manual feature engineering.

The DLVPM method is extremely versatile. The measurement model formula, ${\bar{Y}}_{i}({X}_{i},\,{U}_{i},{W}_{i})$, hides a high level of generality and complexity. In practice, almost any kind of neural network can be used here. This means that the method can be used to create embeddings shared by feed-forward networks, convolutional networks and transformers and so on, where architectural choices will depend on the data under analysis.

We introduce two different formulations of DLVPM, using different orthogonalization procedures. During training, the orthogonalization constraint is achieved via whitening or iterative orthogonalization. Whitening is a widely used approach in deep learning. Iterative orthogonalization has the advantage that it prioritizes DLVs by their importance—a feature of considerable importance in the biological application presented here.

### DLVPM-Twins

Although DLVPM is primarily designed to uncover associations between multiple data types, it also excels at discovering useful representations of a single data type. In this context, DLVPM mirrors the objectives of confirmatory factor analysis^28^ within classical path modelling—each serves to distill complex data into simpler, interpretable structures. However, although confirmatory factor analysis confines itself to linear relationships, DLVPM extends this capacity into the nonlinear domain by enabling the use of deep neural network architectures. When used in this manner, DLVPM falls into the class of methods called Siamese or twin networks^29^. This class of methods has become popular across a wide range of fields in recent years^30,31^. Using this type of method, two augmented (distorted) versions of the same input are passed to a network. The model is then trained to learn features invariant to the applied augmentations, thereby promoting the development of robust and generalizable features (Fig. 1b). The optimization criteria for this method can be written as

$$\max_{U,W}{\rm{tr}}({\bar{Y}}_{A}{({X}_{A},U,W)}^{{\rm{T}}}{\bar{Y}}_{B}({X}_{B},U,W))$$

subject to the constraint

$${{\bar{Y}}_{A}}^{{\rm{T}}}{\bar{Y}}_{A}=I\,{\rm{and}}\,{{\bar{Y}}_{B}}^{{\rm{T}}}{\bar{Y}}_{B}=I,$$

where ${\bar{Y}}_{A}$ and ${\bar{Y}}_{B}$ are outputs of the neural network with weights *U* and *W*. Here *X*_*A*_ and *X*_*B*_ are different augmentations of the same input *X*. As was the case for the full DLVPM path-modelling procedure, both whitening and iterative orthogonalization schemes were used to impose orthogonality.

**Extended Data Fig. 1:**
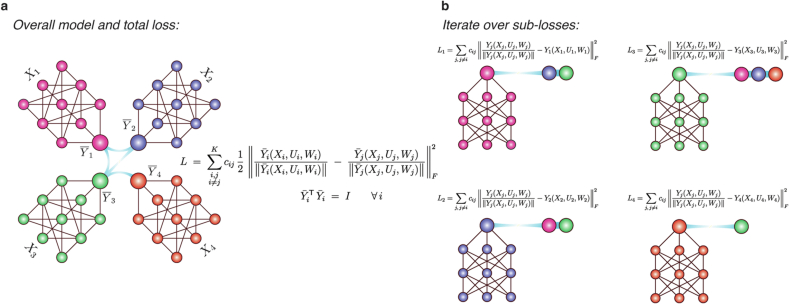
Illustration of the iterative mechanism underpinning the DLVPM algorithm. **a:** Shows the overall model. The aim of the method is to maximize the sum of correlations between deep latent variables (DLVs) connected by the path model. This optimization can be achieved by minimising a sum of least squares losses between the output of each measurement model, and the measurement models they are connected to via the path model. **b**: The overall loss can be minimized by iteratively minimizing least squared losses between the output of each measurement model, with outputs from connected measurement models.

A full and robust mathematical formulation of DLVPM is given in the Methods. The algorithm is illustrated further in Extended Data Fig. 1.

### Confounding effects

**Extended Data Fig. 2:**
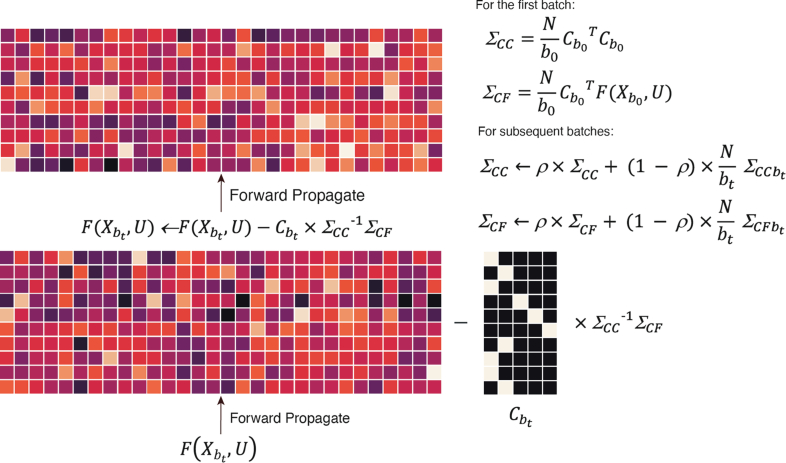
Illustration of the confound removal algorithm. This figure illustrates the batch-wise confound removal process, where confounding variables are accounted for and orthogonalized in the penultimate layer of the model to ensure that the final output is independent of these variables. The first step involves calculating and subtracting the confound influence from the batch signal, followed by the propagation of the corrected signal through the network. Mathematical definitions of the variables shown here are given in the Online Methods.

Previous research has highlighted how factors such as the acquisition site can undermine the replicability and generalizability of studies on molecular and histological data^32^. To address these issues, we implemented an approach for controlling the effect of confounders within a custom neural network layer. This layer uses the Moore–Penrose pseudo-inverse of a matrix of nuisance covariates to remove the effect of confounding variables. We used this method to remove confounding effects of site in all the DLVPM analyses. In particular, this versatile layer represents a separate contribution from the main DLVPM method, and can be used in any neural network model. The mechanics of this approach are thoroughly detailed in the Methods and are illustrated in Extended Data Fig. 2.

### Benchmarking DLVPM-Twins

**Fig. 2:**
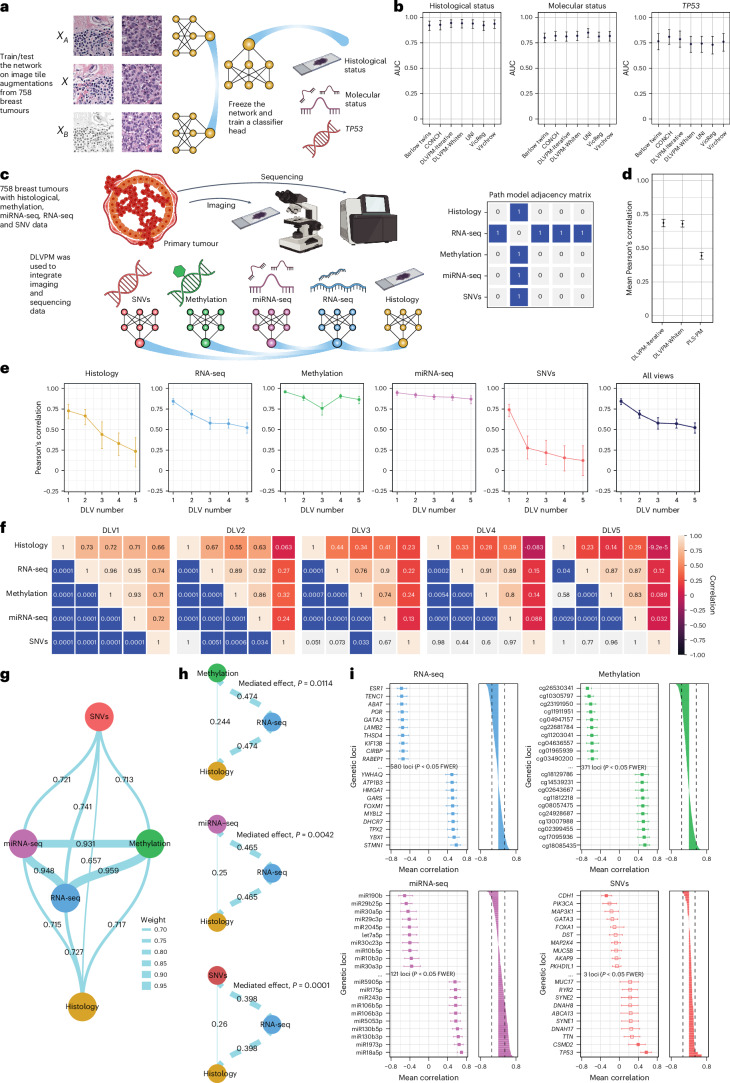
Results of DLVPM analysis applied to TCGA breast cancer data. **a**, Illustration of DLVPM in a Siamese/twin network configuration. **b**, Plots comparing the performance of DLVPM-Twins against VicReg, Barlow twins and several pretrained foundation models (*n* = 152). The error bars represent the mean-centred 95% bootstrapped confidence intervals. **c**, Illustration of the DLVPM method showing a graph representation of the path model, and the associated adjacency matrix. **d**, Comparison of the mean Pearson’s correlation across dimensions and data modalities for DLVPM and PLS-PM (*n* = 152). The error bars represent the mean-centred 95% bootstrapped confidence intervals. **e**, Plots show the mean Pearson’s correlation of each DLV, with DLVs from the data types connected by the path model. The error bars represent the mean-centred 95% bootstrapped confidence intervals (*n* = 152). **f**, Association matrices for all the five DLVs. The entries in the top triangular part of the matrix indicate the Pearson’s correlation values between the different data types. The entries in the bottom part of the matrix are significance values for these correlations, obtained using permutation testing (*n* = 152). **g**, Path model linking the omics and imaging data types included in this analysis. This graph represents the first orthogonal mode of variation between DLVs. The edges connecting the network nodes are labelled with Pearson’s pairwise correlation coefficient (*n* = 152). **h**, Results of mediation analyses carried out using the first DLV. The numbers on the network graph are beta values. The significance of the mediated effect is shown on the right of the graph (*n* = 152). **i**, Results of additional analyses to localize effects to particular genetic loci. The plot shows the Pearson’s correlation values between the genetic loci and DLVs connected to the data view under analysis by the path model. The plots on the left show the ten most positively and negatively associated genetic loci for each data type. The error bars represent the mean-centred 95% bootstrapped confidence intervals. The bar plots show the Pearson’s correlation values for all the loci under analysis, along with the family-wise error-corrected (FWER) significance threshold (*n* = 152). Panels **a** and **c** created with BioRender.com.

In the past couple of years, a number of large-scale ‘foundation models’ have been trained on histological images from cancer^33,34^. Foundation models are trained in a self-supervised manner to learn meaningful representations of the histological input data, which can then be leveraged for various applications. Although DLVPM is primarily designed to learn dependencies between different data types, DLVPM-Twins can be used to pretrain a histological model, which can be used in downstream tasks. DLVPM-Twins is trained by passing a network augmented versions of the same input (Fig. 2a). In the present context, this means flipping, rotating and altering the colour of images passed to the network. The model is then trained to learn orthogonal DLVs that are invariant to these distortions. This encourages the network to learn meaningful representations of data.

The performance of self-supervised methods/foundation models is typically compared on outcome-driven tasks. We compared the performance of a model trained using DLVPM-Twins with models trained using VicReg and Barlow twins, two commonly used Siamese/twin network approaches. We also compared performance with several recently published histological foundation models^33–35^. We benchmarked the performance of different models/methods on several classification tasks: prediction of histological and molecular status, and the presence/absence of *TP53* mutations. This was achieved by training a single-layer classification head on top of embeddings generated by these different procedures. We used 80% of the TCGA breast cancer dataset for training, with the remaining 20% used for testing (*n* = 606 training and *n* = 152 testing; Fig. 2a). Population characteristics of this sample can be found in Supplementary Tables 1 and 2. This train/test split was also used for the full DLVPM path-modelling analysis described in the next section. We trained Siamese models based on the EfficientNetB0 convolutional architecture using DLVPM-Twins, VicReg and Barlow twins (Methods).

We found that the performance of DLVPM-Twins equalled both large-scale foundation models and Barlow twins and VicReg procedures (Fig. 2b). Our method has the advantage over VicReg and Barlow twins methods in that its loss does not require any hyperparameters other than the size of the final embedding. Fewer hyperparameters simplify the training process and increase the model robustness by reducing the need for extensive tuning. Among the two DLVPM variants, the version using iterative orthogonalization has the major advantage that it ranks the DLVs according to the strength of their associations across data types, in a manner akin to the ranking of principal components on the basis of the variance they account for within a dataset. Barlow twins is limited to learning a representation of a single data type; VicReg can learn representations of two data types but it is unclear how the method can be generalized further than this. By contrast, DLVPM can be used to integrate data from arbitrarily many data types.

Our model matches the performance of UNI, Vichrow and Conch, notable foundation models in histology, while maintaining a substantially lower parameter count. Both UNI and Vichrow are trained using the DinoV2 algorithm, which requires the use of a vision transformer. By contrast, our DLVPM-Twins algorithm is more versatile, supporting a variety of neural network models beyond vision transformers. For example, we have effectively trained an EfficientNetB0, a convolutional neural network, using this method. This flexibility allows DLVPM-Twins to adapt to different network architectures, providing a strong advantage for applications across a diverse range of datasets and computational constraints.

### Full DLVPM

Next, we trained DLVPM for the purposes of full path modelling. We applied DLVPM to data from 758 breast cancer samples from TCGA. Our initial goal was to uncover relationships across five data types: histological images, single-nucleotide variants (SNVs), methylation profiles, microRNA (miRNA) sequencing (miRNA-seq) and RNA sequencing (RNA-seq) expression data. We positioned the transcriptomic data at the centre of the path-modelling analysis, recognizing its pivotal role in mediating the effects of genomic and epigenomic changes on histological tissues through gene expression modulation. Using this path model, all other data types are linked to one another indirectly through the RNA-seq data (Fig. 2c). Training and testing were carried out using the same 80%–20% train–test split as for the DLVPM-Twins analysis.

**Extended Data Fig. 3:**
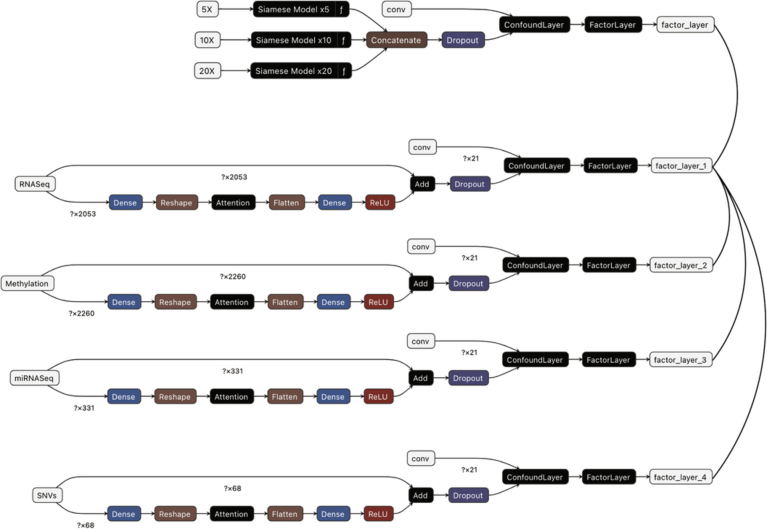
DLVPM path model used to combine multiomic and imaging data. The figure illustrates the neural network models used as measurement models for each data type in the present investigation. The figure shows the neural network used to process histology data. There are three inputs to the network. These inputs take imaging data that has been tiled, passed through a pre-trained DLVPM-Twins model, and then mean averaged over the network output for each tile. Each of the omics data modalities was processed with a residual network that combines an attentional mechanism with the raw input via a skip connection.

We must also specify the measurement models for each data type; these models define the manner in which data are processed and connected. For the histology data, we specified a neural network that aggregates effects arising at different magnifications. Here it takes DLVPM-Twins models trained at ×5, ×10 and ×20 magnifications, each utilizing the EfficientNetB0 convolutional architecture^36^. For the genetic data, we used a residual network with an attentional mechanism. This allows the neural network to aggregate the linear effects from individual genes, with interaction effects between genes. The full neural network encompassing the path model and individual measurement models is shown in Extended Data Fig. 3.

Following model training, we compared the performance of DLVPM with PLS-PM^23^. PLS-PM has an identical objective to DLVPM, but is only able to model linear effects. In this comparison, both iterative orthogonalization and whitening versions of DLVPM demonstrated greatly superior performance compared with PLS-PM (Fig. 2d). Comparing performance with other deep learning methods for multimodal data integration is not feasible in this manner, as fundamentally different purposes are encoded by distinct loss functions unique to each method. As previously noted, the DLVPM variant utilizing iterative orthogonalization has the advantage that it ranks DLVs by their importance. For this reason, we used the results from this approach in all subsequent analyses. This ranking of DLVs by their mean association is shown in Fig. 2e for the model as a whole and for each data type individually.

Next, we evaluated the specific associations the method uncovers between data types. These associations and their permutation family-wise error-corrected significance levels are shown in Fig. 2f. This analysis uncovers multiple orthogonal paths that connect molecular and histological data. A network graph illustrating the DLVPM path model for the first set of DLVs is illustrated in Fig. 2g.

**Extended Data Fig. 4:**
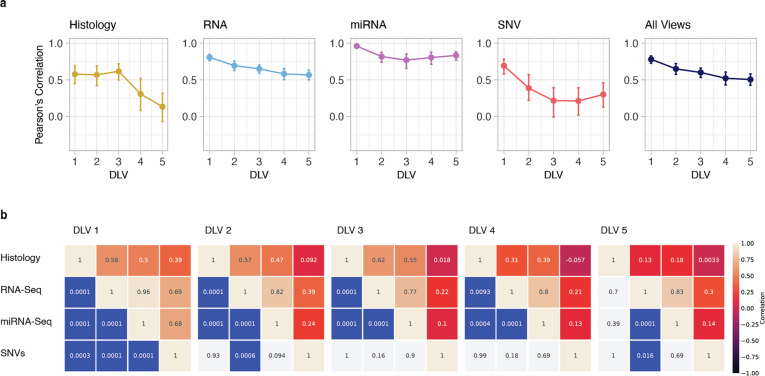
Replication of a TCGA DLVPM model in CPTAC data. **a:** For each data type, these plots show the mean Pearson’s correlation of each DLV, with DLVs from data types connected by the path model, in the CPTAC dataset (n = 105). The error bars on the plot denote mean-centred 95% bootstrapped confidence intervals. **b**: Association matrices for all five DLVs. Entries in the upper triangular part of the matrix indicate Pearson’s correlation values between different data-types. Entries in the lower part of the matrix are significance values for these correlations, obtained using permutation testing.

To ensure the robustness of the out-of-sample results, we also carried out a fivefold cross-validation analysis, in place of the single train–test split used here. Correlations were of a similar magnitude to the main results, confirming robustness (Supplementary Fig. 1). We further replicated our main results on 105 independent patient samples from the Clinical Proteomic Tumor Analysis Consortium (CPTAC) project^37,38^. The CPTAC data allowed us to validate the robustness of DLVPM across datasets, demonstrating similar patterns of associations between different data modalities (Extended Data Fig. 4).

**Extended Data Fig. 5:**
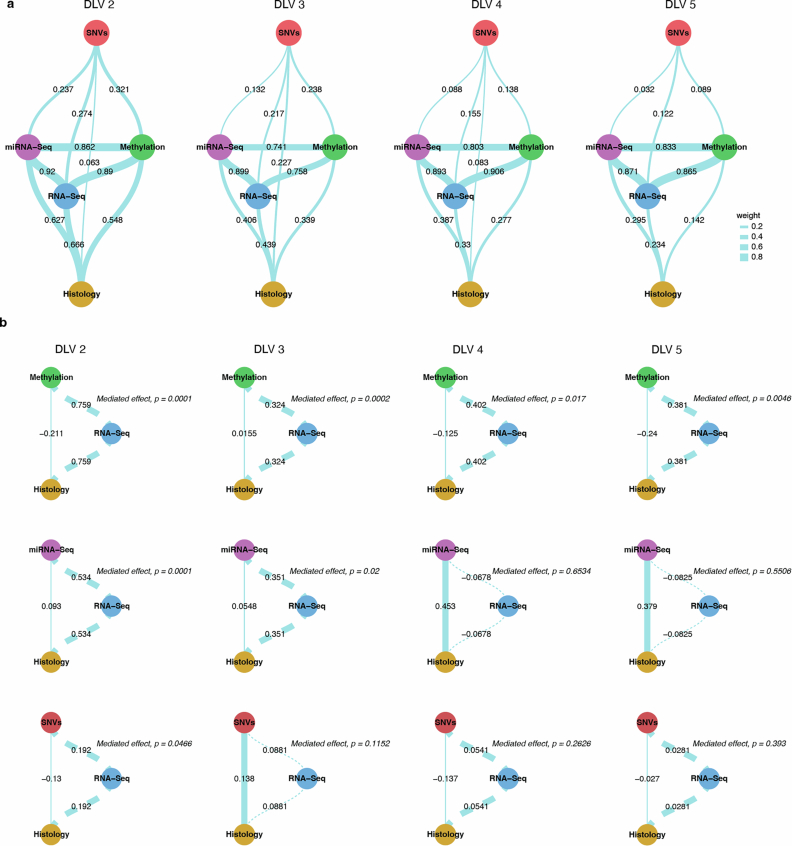
DLVPM path models in TCGA data. **a:** This figure panel shows DLVPM path models for all DLVs after the first, trained and tested on TCGA data. Each of these network graphs represents an orthogonal associative mode linking different data, established using the DLVPM procedure (n = 152). **b**: These network graphs illustrate different multiomic mediation analyses using DLVs estimated using DLVPM. In each case, DLVs from the histology data take the place of dependent variables. DLVs from the RNA-Seq data act as mediator variables. The significance of direct and mediation effects of DLVs derived from: SNVs, miRNA-Seq and Methylation data were all tested in mediation models. Straight paths between the independent variable and the dependent variable represent direct effects, where the width of the edge linking them is equal to the beta value for the direct effect in the mediation model. The indirect effect is represented by the path through the RNA-Seq DLV. Significance values were obtained using bootstrapping (n = 152).

A major strength of DLVPM is its ability to uncover and analyse indirect effects, such as mediation relationships among variables, opening up the possibility of investigating the intricate dynamics that define complex systems. We examined how RNA-seq DLVs mediate the interaction between various genetic and epigenetic variables (specifically methylation, miRNA-seq and SNVs, which are treated as independent variables) and histological outcomes, which are treated as dependent variables. Path diagrams (Fig. 2h) visually depict these mediation processes, highlighting both direct effects and those mediated via the RNA-seq DLV. These analyses highlight the crucial role of gene expression in linking genetic and epigenetic changes to cellular and tissue-level phenotypes, offering insights into the complex interactions that drive histological changes (Fig. 2h and Extended Data Fig. 5).

**Extended Data Fig. 6:**
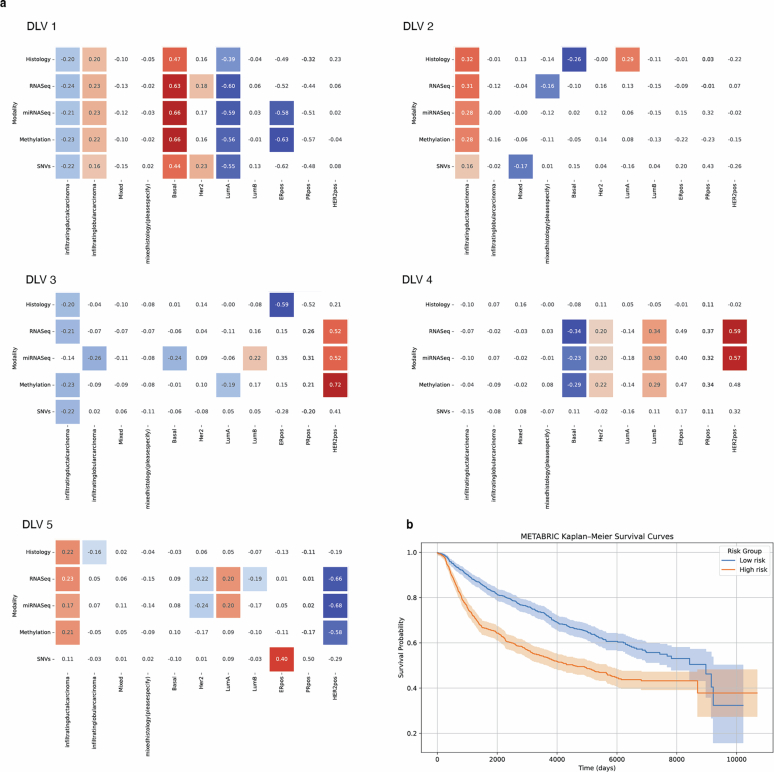
Clinical analyses in TCGA data. **a:** The heat maps shown in this figure illustrate associations between DLVs, estimated using the full DLVPM model, and clinical molecular and histological characteristics. Quantitative values in each heatmap are the Pearson’s Correlation Coefficient between different clinical types, and DLVs. Heatmap squares that are coloured are significant at the p < 0.05 FWER corrected level. Squares that are not coloured are non-significant (n = 152). **b**: Kaplan–Meier survival curves for patients stratified into high- and low-risk groups based on risk scores derived from a Cox proportional hazards model n the METABRIC dataset, stratified on RNASeq and SNV DLVs. Patients were divided into high- and low-risk groups based on the median risk score: those with scores above the median were classified as ‘High Risk,’ while those with scores below the median were classified as ‘Low Risk.’ The x-axis represents progression-free interval (PFI) in days, and the y-axis represents survival probability. Shaded regions indicate confidence intervals (n = 1980).

Consistent with DLVPM’s path model, which links all data types through the RNA-seq data, we observed that all DLVs—even those originating from the histology data—demonstrated a stronger association with established clinical molecular subtypes than with histological types. For instance, the first DLV distinctly stratified basal and luminal molecular subtypes across all data modalities (Extended Data Fig. 6a).

We used Cox proportional hazards regression to predict the progression-free interval from all the DLVs for both DLVPM-Iterative (concordance index (CI) = 0.65, *P* = 0.26, *n* = 152) and DLVPM-Whiten (CI = 0.64, *P* = 0.27, *n* = 152). Neither results were significant. However, the utility of TCGA for survival analysis is limited by its short follow-up period (mean follow-up, 3.4 years), which constrains the study of long-term outcomes. Additionally, the analysis is underpowered as full generality requires that we only use the test set (152 patient samples) for outcome prediction. To overcome these limitations, we recalculated the SNV and RNA-seq DLVs using data from the METABRIC study (Methods), which features a much longer follow-up; the longer follow-up period (mean follow-up, 9.29 years) in METABRIC confirmed that both DLVPM-Iterative (CI = 0.61, *P* = 1.4 × 10^−14^, *n* = 1,980) and DLVPM-Whiten (CI = 0.61, *P* = 5.4 × 10^−14^, *n* = 1,980) are strongly predictive of clinical outcomes (Extended Data Fig. 6b). We benchmarked the performance of DLVPM against other widely used methods for multimodal data integration, including PLS-PM^23^, MOFA+^39,40^ and a multimodal autoencoder^41^ (Methods). DLVPM demonstrated superior predictive performance to PLS-PM and the multimodal autoencoder, and similar predictive capabilities to MOFA+ (Supplementary Table 3).

**Extended Data Fig. 7:**
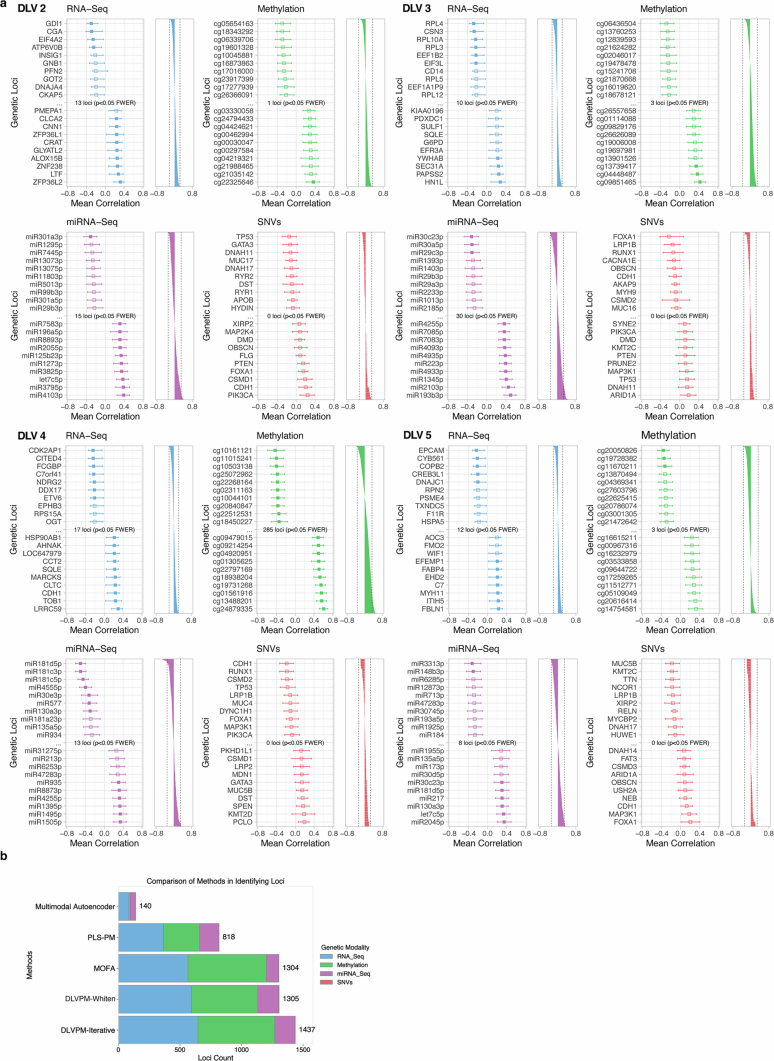
Results of additional analyses to localize effects to particular genetic loci. **a:** The plot shows association values between genetic loci, and DLVs connected to the data-view under analysis by the path model. The panel on the left shows the ten most positively and negatively associated genetic loci. The error bars on the plot denote mean-centred 95% bootstrapped confidence intervals. The panel on the right shows all genetic loci under analysis along with the significance threshold cut-off (n = 152). **b**: The total number of multiomic loci identified as showing an individually significant association with latent variables estimated from different data-integration methods (n = 152).

DLVPM operates fundamentally as a multivariate approach, designed to uncover factors exhibiting high correlation across diverse data types, including genetic and imaging datasets. The multiomic DLVs constructed by the model represent complex polygenic factors. Owing to its multivariate nature, the method initially precludes the direct attribution of significance to specific genetic loci within the model. To bridge this gap, we ran additional analyses to isolate genetic/epigenetic loci that demonstrate significant correlations individually with DLVs (Methods). Each DLV produces a stratification of imaging/multiomic subtypes, with loci exhibiting either positive or negative associations. Hundreds of loci made individually significant contributions to the DLVPM model (Fig. 2i, Extended Data Fig. 7a and Supplementary Table 4). Permutation testing using the distribution of the maximal statistic was used to control for multiple comparisons and provide strong control over the family-wise error rate.

The first DLV shows the strongest associative mode linking the omics and imaging data, and effects on histology are mediated via gene expression, quantified by RNA-seq. This prompted us to focus our initial interpretation of individually significant loci on RNA-seq data from DLV 1. First, we investigate negatively associated loci: this path model stratifies genes important in luminal–basal transcriptional differentiation program. *ESR1*, whose protein defines the luminal subtype, is used for the clinical diagnosis of breast cancer, and is a target in hormone therapy^42^. Furthermore, *GATA3* encodes a transcription factor that regulates luminal cell differentiation and exhibits a shift from a tumour-suppressing role to a tumour-promoting role in breast cancer via the deregulation of *THSD4* (ref. ^43^), which also shows a strong negative association. *PGR*, which encodes the progesterone receptor and is crucial for prognosticating hormone treatment outcomes in breast cancer, is also significant here and is closely linked to luminal breast cancer^44^. By contrast, genes showing a strong positive association with DLV 1 have been primarily linked to the basal breast cancer subtype. *STMN1* encodes a protein that has been implicated in cell cycle progression and mitosis and has been investigated as a therapeutic target in breast cancer^45^. *YBX1* encodes a protein strongly implicated in breast cancer, and is particularly noted for its role in cell migration and invasion^46^, as well as drug resistance^47^. *TPX2* overexpression has also been linked to more aggressive forms of breast cancer^48^. *MYBL2* encodes a protein that has been shown to drive cell cycle progression in breast cancer^49^. A luminal–basal stratification on the first DLV was supported by gene set enrichment analysis (GSEA) carried out between DLVs and gene expression scores (Methods); other DLVs were associated with different cancer-related processes (Supplementary Fig. 2).

We again benchmarked the performance of DLVPM against other widely used methods for multimodal data integration (Methods), in the task of identifying individually significant genetic loci. We found that DLVPM outperformed these methods in this task, with DLVPM identifying the most genetic loci associated with the multimodal data integration model (Extended Data Fig. 7b).

### Characterization of histological data

A number of important previous studies have leveraged histological data to predict clinical molecular status, detect the presence or absence of known oncogenic mutations, and delineate bulk transcriptomic profiles of tumours using deep neural networks^11,50^. Although these studies are hugely important, they largely operate within the confines of pre-existing hypotheses. The DLVPM methodology stands out for its capacity to unearth previously unrecognized relationships across diverse data modalities.

**Fig. 3:**
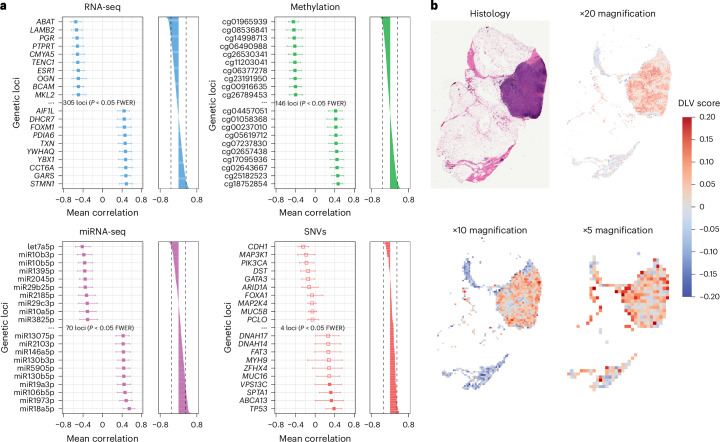
Relations between multiomic loci and histology in TCGA breast cancer data. **a**, Results of additional analyses to localize effects to identify omics loci showing an individually significant association with the histological data. The plot shows Pearson’s correlation values between genetic loci, and the first histological DLV. The plots on the left show the ten most positively and negatively associated genetic loci for each data type (*n* = 152). The error bars represent the mean-centred 95% bootstrapped confidence intervals. The bar plots show Pearson’s correlation values for all the loci under analysis, along with the family-wise error-corrected significance threshold. Owing to space limitations, we only show the analyses for the first DLV here. **b**, Normalized heat maps for a tumour, on the first DLV, at ×5, ×10 and ×20 magnifications.

**Extended Data Fig. 8:**
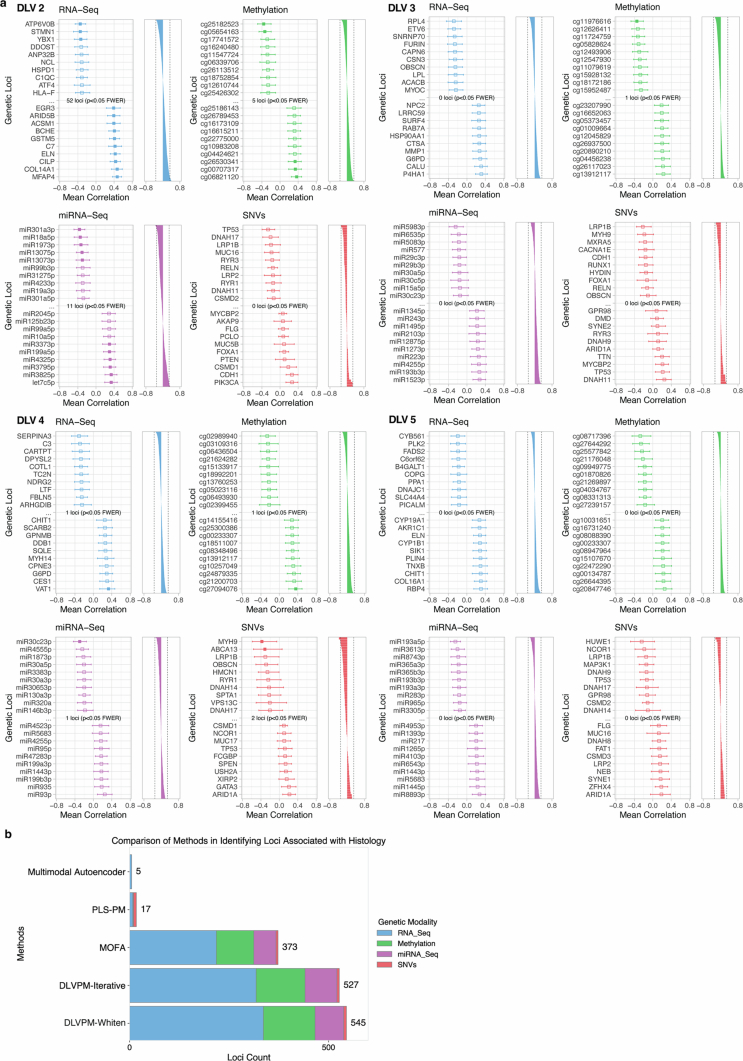
Results of additional analyses to identify genetic loci associated with histology. **a:** The plot shows association values between genetic loci, and histological DLVs. The panel on the left shows the ten most positively and negatively associated genetic loci. The error bars on the plot denote mean-centred 95% bootstrapped confidence intervals. The panel on the right shows all genetic loci under analysis, along with the significance threshold cut-off (n = 152). **b**: The total number of multiomic loci identified as showing an individually significant association with histological latent variables estimated from different data-integration methods (n = 152).

We ran further analyses focused on pinpointing multiomic loci that show individually significant correlations with histological DLVs, which represent an outcome phenotype. Our investigations not only corroborated existing knowledge by identifying histological–genetic associations with well-documented oncogenes such as *TP53* but also uncovered significant links between histological features and hundreds of previously uncharted multiomic loci (Fig. 3a, Extended Data Fig. 8a and Supplementary Table 5).

The first histological DLV exhibited by far the largest number of individually significant associations with multiomic loci (Fig. 3a, Extended Data Fig. 8a and Supplementary Table 5). Many of the genes making the strongest individual contribution to the overall model were also amongst those showing the most pronounced correlations with the histology data: *PGR* showed a strong negative association with DLV 1; the expression of this gene has been reported as exhibiting pronounced inverse correlations with histological grade, mitotic rate and nuclear pleomorphism in hypothesis-driven studies involving expert pathological assessment^51^. The *ABAT* gene, which has been previously linked to ER-positive breast cancers^52^, shows the strongest negative association with DLV 1. Genes showing a strong positive association with histology are primarily associated with the more aggressive basal type. The products of the genes *TPX2*, *ANP32B*, *PFKP*, *CHI3L1*, *S1009A*, *FOXM1*, *STMN1* and *MYBL2* are crucially involved in cellular proliferation^45,49,53–58^. This is particularly noteworthy in this context where increased cellular proliferation will result in visible changes in tumour grade and mitotic rate.

We benchmarked the performance of DLVPM against other widely used methods for multimodal data integration, in the task of identifying individually significant genetic loci associated with histology data. We found that DLVPM outperformed these methods in this task (Extended Data Fig. 8b).

As was previously noted, we trained our model on small tissue sections known as image tiles (Methods). Once the DLVPM model is trained, we can deconvolve tile-wide effects back into the image space. We used a neural network model that takes tiles at ×5, ×10 and ×20 magnifications (Extended Data Fig. 3). Figure 3b shows heat maps at each of these magnifications, for DLV 1. Histological effects and their molecular concomitants are explored in greater detail later in the Article.

### Single-cell characterization

DLVPM acts to simultaneously integrate multiomic and imaging data, and reduce their dimensionality. This results in a compressed representation of important genetic and physiologic processes in cancer into a small number of DLVs. Once the overall model is trained, the individual measurement submodels can be used to help further characterize the model, and obtain deeper biological insights. For this purpose, we applied the trained DLVPM model to single-cell, cell-line and spatial transcriptomic data.

Cancer cells are the fundamental units of neoplastic disease. Tumours are composed of a diverse array of cancer and stromal cells, with distinct genetic and phenotypic properties. We applied the RNA-seq component of the full DLVPM model, trained on TCGA, to data from the single-cell breast cancer encyclopaedia, which contains RNA-seq data from 100,064 single cells^59^. This allows us to determine individual cell types that contribute significantly to each DLV, providing increased phenotypic resolution and potential insight into heterotypic interactions between cells that are typical of tumours scoring highly on different DLVs.

**Fig. 4:**
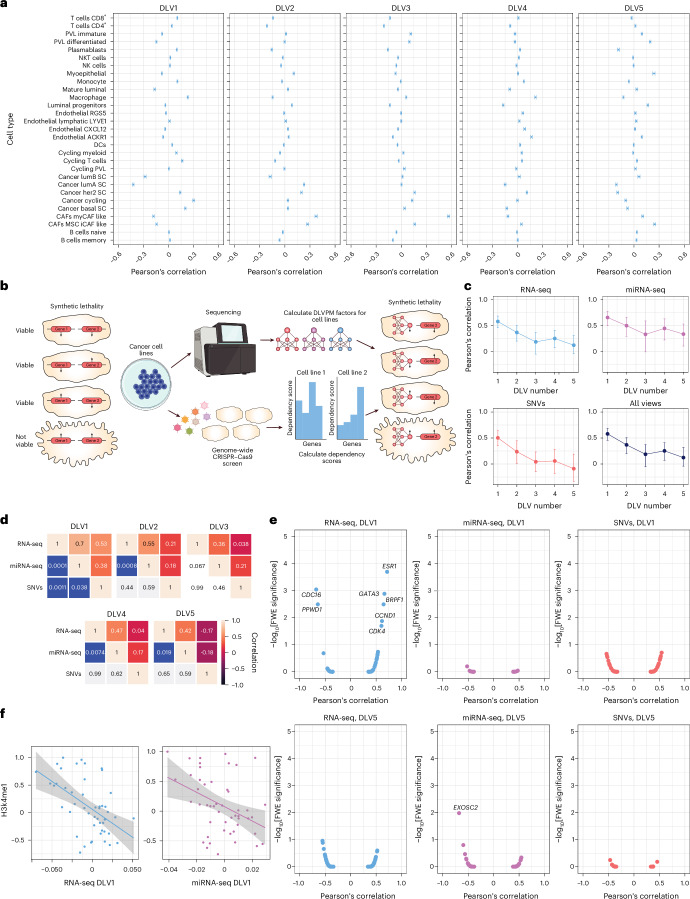
Results from analyses of a DLVPM model trained on TCGA data and applied to single-cell and cell-line data. **a**, Stratification of single cells based on the DLVPM model, applied to their transcriptomic profiles. The error bars show the mean-centred 99.9% bootstrapped confidence intervals. DCs, dendritic cells; MSC, mesenchymal stem cell; NK, natural killer; NKT, natural killer T; PVL, perivascular‐like. **b**, Conceptual illustration showing the general principle of synthetic lethality. The up and down arrows associated with the genes represent different states, for example, mutated/non-mutated. Genetic features are synthetically lethal if the cell viability is affected when they both take on a particular state in the cell. Here, this is represented by the up arrows. The right part of the panel shows how DLVPM can be used to uncover new genetic vulnerabilities using this principle. DLVs are constructed from the sequencing data, and are used to predict susceptibility to gene knockout. **c**, For each data type, these plots show the mean Pearson’s correlation of each DLV, with DLVs from data types connected by the path model. The error bars on the plot denote the mean-centred 95% bootstrapped confidence intervals (*n* = 61 for RNA-seq, *n* = 67 for SNVs, *n* = 50 for miRNA-seq). **d**, Association matrices for all the five DLVs. The entries in the top triangular part of the matrix indicate the Pearson’s correlation values between the different data types. The entries in the bottom part of the matrix are significance values for these correlations, obtained using permutation testing (*n* = 61 for RNA-seq, *n* = 67 for SNVs, *n* = 50 for miRNA-seq). **e**, Magnitude of associations between DLVs and CRISPR–Cas9 gene dependency scores, against their family-wise error-corrected significance levels. The labelled genes are those with significance levels under *P* = 0.05. We only show the volcano plots in which there was a significant association between the DLVPM variable and the CRISPR–Cas9 data (*n* = 42 for RNA-seq, *n* = 45 for SNVs, *n* = 34 for miRNA-seq). **f**, Associations between the first RNA-seq DLV and first miRNA-seq DLV, and the histone modification H3K4me1 (*n* = 49 for RNA-seq, *n* = 49 for miRNA-seq). Data are presented as linear regression lines (centre) with 95% confidence intervals (error bands). Panel **b** created with BioRender.com.

In concordance with earlier results, the first DLV exhibits strong negative enrichment for luminal cells types; this DLV also shows strong positive enrichment for basal and cycling cancer cells, which indicate a more aggressive molecular type (Fig. 4a). Interestingly, DLV 3 shows extremely strong positive enrichment for myofibroblastic cancer-associated fibroblasts (myCAFs), a subtype of CAFs identified by their expression of alpha-smooth muscle actin, which contributes to their effect on the tumour microenvironment, affecting tissue stiffness, cancer cell invasion and immune suppression, making them an important marker for aggressive cancer types^60^. Results from this secondary analysis highlight the capability of the DLVPM model to elucidate complex cellular interactions within tumours, enhancing our understanding of cancer cell dynamics and tumour heterogeneity.

### Cancer-cell-line characterization

To further evaluate the utility of the DLVPM model, we applied it—having been trained on TCGA patient data—to multiomic cell-line data from the Cancer Cell Line Encyclopedia (CCLE). Our objective was to explore if breast cancer cell lines, stratified on the basis of DLV profiles, exhibited differential sensitivity to genome-wide knockouts, facilitated by CRISPR–Cas9 loss-of-function screens. This approach not only promises to enhance our understanding of the biological importance of each DLV but also identifies potential therapeutic targets by pinpointing gene knockouts that exhibit synthetic lethal interactions in specific cancer contexts. This analysis utilizes data from the cancer dependency map^61^. A schematic of the analysis is shown in Fig. 4b.

We first tested if associations between omics DLVs specified by the DLVPM model trained on the TCGA patient data replicated in the cell-line data from the CCLE. We found that the first four DLVs retained significant associations (Fig. 4c), with correlations between RNA-seq and miRNA-seq DLVs exhibiting a particularly large effect (Fig. 4d).

We then conducted analyses to identify synthetic lethal interactions between DLVs and genome-wide CRISPR-Cas9 dependency scores. Without using any biologically informed priors, and using a model trained on patient rather than cell-line data, we identified several genes that are already targets of frontline therapies in breast cancer (Fig. 4e). These associations were linked to DLV 1: as previously noted, *ESR1* encodes an oestrogen receptor and ligand-activated transcription factor. The protein encoded by this gene regulates the transcription of many oestrogen-inducible genes involved in growth, metabolism, gestation and sexual development. Endocrine therapy to inhibit oestrogen is an extremely important therapy for oestrogen-receptor-positive breast cancers^42^. The first RNA-Seq DLV also shows a dependence on both the cyclin-dependent kinase-encoding gene *CDK4* and its regulator *CCND1*. Higher *CCND1* expression has been linked to an increased risk of death in ER+ breast cancer^62^. Drugs designed to inhibit the action of *CDK4* protein products have recently been shown to improve prognosis in hormone-dependent cancers, beyond the use of endocrine therapy alone^63^. *GATA3* showed a very high synthetic lethal dependency with DLV 1 in this cell-line data. As previously noted, the *GATA3* protein has been shown to be crucial in the development of the mammary gland, and is critical to the luminal cell program in the breast^64,65^. *GATA3* encodes a protein that acts as a pioneer factor, a special type of transcription factor that can bind directly to chromatin. Pioneer factors have been called the master regulators of the epigenome and of cell fate^66^, which operate by opening previously inaccessible regulatory elements. These factors have the largest effect on transcription via histone modification and chromatin remodelling^66^. We calculated the association between the first RNA-seq and miRNA-seq DLVs and the global chromatin profile, which showed a strong link to the histone modification H3K4me1. For RNA-seq, this association was *r* = –0.49 (*P* = 1.74 × 10^−4^, *n* = 49); for miRNA-seq, *r* = −0.40 (*P* = 0.0063, *n* = 49; Fig. 4f).

Cells with low scores on DLV 1 were susceptible to the knockout of a wholly different set of genes (Fig. 4e). The strongest dependency relation was with *CDC16*; this gene is part of the APC/C (anaphase-promoting complex, also known as the cyclosome), which governs exit from mitosis^67^. *PPWD1* is thought to be involved in protein folding^68^, but its role in cancer is less well studied.

Cells scoring low on the miRNA-seq component of DLV 3 were susceptible to the knockout of *EXOSC2*. The overexpression of *EXOSC2* has been previously shown to promote breast cancer cell growth, migration, angiogenesis and tumour formation, whereas its knockdown reduces these effects^69^.

**Extended Data Fig. 9:**
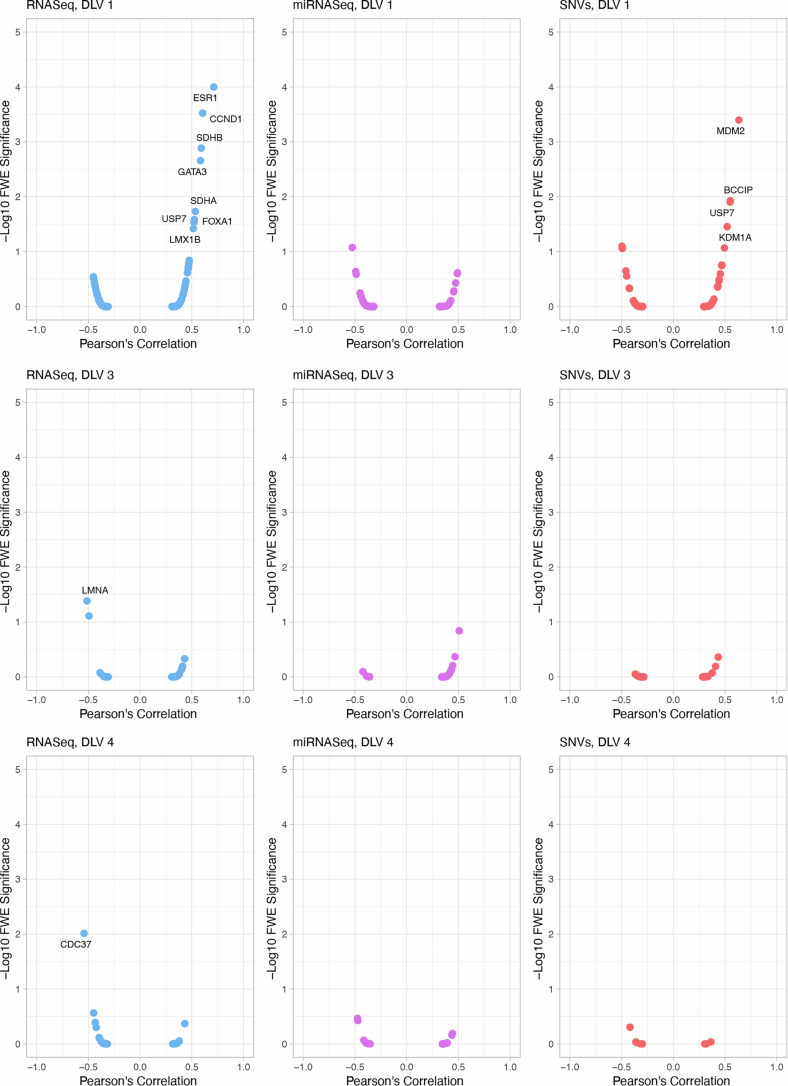
Associations between DLVs and RNAi gene dependency scores. These figures show plots of pairwise association of DLVs against -log10 p-values, plotting the relation between DLVs and gene dependency scores derived from RNAi data (n = 58 RNA-Seq, n = 61 SNVs, n = 49 miRNA-Seq). We only show the volcano plots where there was a significant association between the DLVPM variable and the RNAi data.

We repeated these analyses using an RNAi loss-of-function screen and obtained largely similar results (Extended Data Fig. 9). Following multiple comparisons correction, no significant dependency relations were uncovered using individual genetic features, highlighting the benefit of our polygenic, compared with monogenic approaches. In summary, our cancer dependency map analysis underscores that it is possible to identify genes essential to the cellular functioning of certain molecular subtypes of breast cancer using a DLVPM model trained on patient data. Our findings suggest that DLVs can serve as biomarkers for identifying cell lines that are particularly susceptible or resistant to specific genetic interventions, underscoring the potential for DLVPM-guided targeted therapies in oncology.

### Spatial transcriptomics

Our analyses using the DLVPM model identifies histological concomitants of polygenic modes of variation in cancer, and their synthetic lethal genetic dependencies. Spatial transcriptomic data offer a more spatially resolved view of the genetic basis of aberrant changes in tissue structure. We found the genes *ESR1*, *GATA3* and *CCND1* to be highly dependent on DLV1 based on gene dependency scores from CRISPR–Cas9 screens. Each of these genes showed an individually significant association with histology (Fig. 3a, Extended Data Fig. 8 and Supplementary Table 5), and are also included as part of the Xenium spatial transcriptomic gene panel^70^. We used the Xenium spatial transcriptomic data to investigate the association between the expressions of these genes, and the histological concomitants of DLV 1 across individual tumours. We assessed tile-wise effects at ×20 magnification, as this resolution is closest to the subcellular Xenium resolution.

**Fig. 5:**
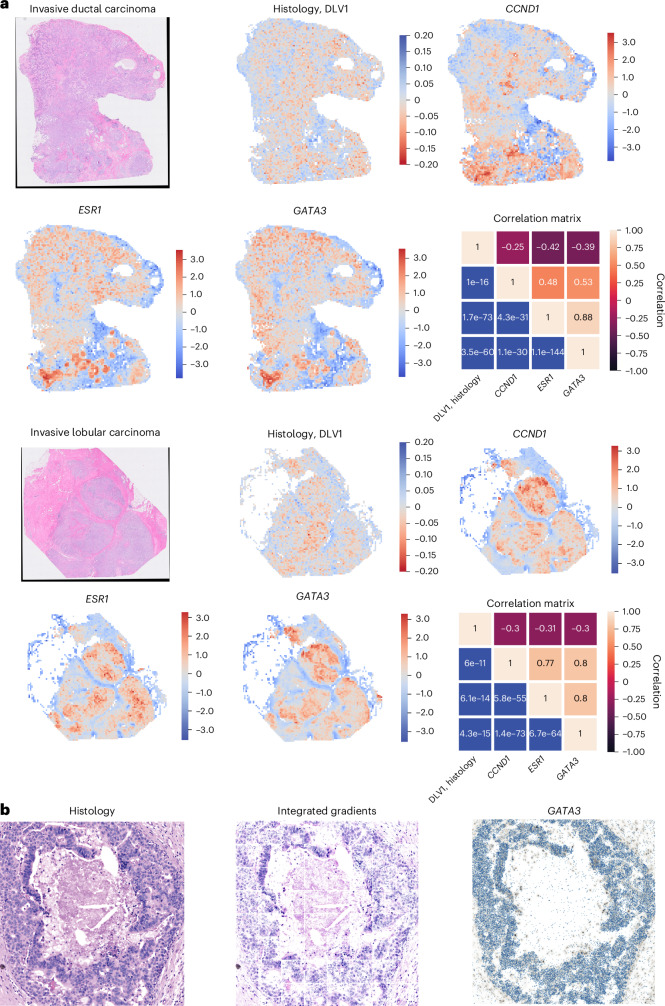
Results from the analyses of a DLVPM model trained on TCGA data and applied to spatial transcriptomics data. **a**, Tile-wise heat maps generated from the DLVPM model, trained on the TCGA data, and applied to histological and associated spatial transcriptomic data. The colour map is flipped for the normalized histology heat map as this DLV shows a negative association with the genes of interest. We applied this analysis to invasive ductal carcinoma and invasive lobular carcinoma. The association/significance matrices on the right show correlations between the genes of interest and the first histological DLV for both tumours. The top triangular part of each matrix is denoted with the Pearson’s correlation coefficient between each gene and the histology data. The bottom triangular part of each matrix denotes the significance level between genes and histology data. **b**, Left, a cancerous breast duct, which scored highly on DLV 1. Middle, a feature attribution map, generated using integrated gradients, illustrating the regions of the tumour that contributed most to the DLVPM model. Right, spatially mapped *GATA3* transcripts.

**Extended Data Fig. 10:**
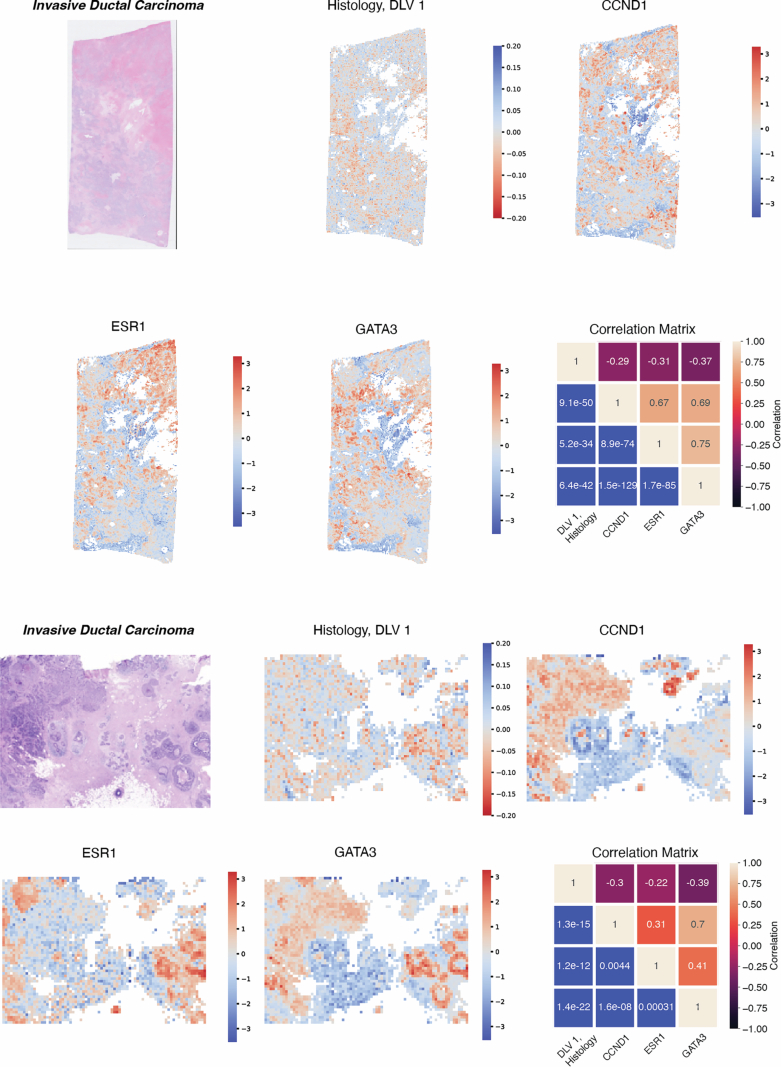
Results from analyses of a DLVPM model trained on TCGA data and applied to spatial transcriptomics data. Tile-wise heatmaps generated from the DLVPM model, trained on TCGA data, and applied to histological and associated spatial transcriptomic data. The colormap is flipped for the histology heatmap as this DLV shows a negative association with the genes of interest. The association/significance matrices on the right show correlations between genes of interest and the first histology DLV for both tumours. The upper triangular part of each matrix is denoted with Pearson’s Correlation Coefficient between each gene, and the histology data. The lower triangular part of each matrix denotes the significance level between genes and histology data.

We found significant associations between the spatial distribution of each of these genes and the histological component of DLV 1 (Fig. 5a and Extended Data Fig. 10) in both invasive ductal carcinoma and invasive lobular carcinoma—the two most common histological types of breast cancer. The strong association between these genes is indicative of their close functional relationship in breast cancer. Each of these genes is the most highly expressed in relatively well-differentiated tumoural regions. This is where the histological component of DLV 1 also scores the lowest. This is consistent with the suggested role of these genes in the early stages of tumour growth and progression.

To pinpoint the histological features that have a pivotal role in linking genetic profiles with histological patterns at a more granular, subtile resolution, we applied the integrated gradients method for feature attribution^71^ (Fig. 5b). This attribution technique assigns importance scores to specific image regions, thereby identifying those that have a pronounced influence on the predictions made by DLV 1. Of particular interest, well-differentiated ductal regions received increased scores, highlighting their marked importance in the model’s determination. Furthermore, these regions show a high concentration of key genetic markers, including *GATA3*, *CCND1* and *ESR1*. The DLVPM analyses carried out on Xenium data forge a connection between the functional essentiality of genes (as assessed by CRISPR–Cas9 loss-of-function screens) and their spatial expression patterns, framing an integrated model of disease pathology.

## Discussion

DLVPM is a method for modelling dependencies between different data types. This method stands out for its ability to uncover complex, nonlinear interactions among both structured and unstructured data types, overcoming the limitations of traditional path-modelling techniques^23^. Initially trained on the extensive TCGA dataset, the modular nature of the method allows for flexible adaptation and further refinement with additional analyses using single-cell, cell-line and spatial transcriptomic data. By applying DLVPM to cancer dependency map data, it unveils critical insights into multiomic dependencies. Furthermore, DLVPM bridges microscopic tissue structure changes with genetic vulnerabilities identified by the same model, illustrating its ability to construct holistic models of illness pathology. This method’s comprehensive data integration capability marks an important step forward, promising applicability beyond cancer to a broad spectrum of diseases.

DLVPM is superior to classical approaches to path modelling in terms of the magnitude of associations the method is able to establish between different data types. This is likely because, in contrast to classical approaches, this method is able to model the complex breakdown in molecular machinery that underpins carcinogenesis. Typically, cancer initiation requires mutations or epigenetic changes in several driver genes. These alterations can affect gene expression at thousands of loci. Many of these genes will be transcription factors, whose purpose is to control the expression of other genes. Classical methods are unable to parse this complexity as they are only able to model linear effects. By contrast, deep learning methods have already been shown to be capable of modelling complex interactions between loci across the genome^12,13^.

Historically, researchers have developed drugs that target oncogenes and block their function. However, not all cancers have oncogenes, which limits the number of possible drug targets. To overcome this challenge, researchers have utilized the principle of synthetic lethality^72^. Synthetic lethal interactions in cancer can be probed using functional genetic experiments such as CRISPR–Cas9 loss-of-function screens. Associations between cells’ molecular features and susceptibility to knockout of a particular gene represent a synthetic lethal interaction. This results in an enormous multiple comparison problem. This approach also fails to respect the intrinsically polygenic nature of cancer as an illness. DLVPM simultaneously integrates the multiomic data it is applied to, and reduces its dimensionality, resulting in a small number of polygenic, multiomic DLVs, avoiding both major pitfalls associated with taking a single-gene approach.

Despite the versatility and power of DLVPM, several limitations warrant careful consideration. First, like other deep-learning-based methods applied in biology, without further analysis or experimental work, results can be difficult to interpret due to the black-box nature of neural networks, which often lack transparency in linking learned features to specific biological processes^73^. This inherent opacity also necessitates special care in validating the results on external datasets. Another weakness of the method is that it requires a full complement of multimodal data. In classical partial least squares (PLS), sophisticated methods for the imputation of missing data have been developed; this is an area for future research^74^. Another technical issue is that the method requires reasonably large batch sizes, an issue common to correlation-based deep learning methods^75^.

DLVPM is implemented in the flexible and user-friendly TensorFlow/Keras ecosystem, enabling the modular construction of complex models tailored to a wide array of data analysis tasks. Using predefined Keras layers, users can define new DLVPM models in just a few lines of code. This modular design not only simplifies the development and testing of sophisticated models but also enhances their extensibility, ensuring that our method can be seamlessly applied across diverse research fields and data types. This toolbox also contains a submodule for confound removal, which can also be used in classification and regression problems, and we anticipate it as being generally useful to the deep learning field.

Many illnesses arise as a result of complex interactions between multiple biological and environmental factors. Several, large, open-access databases, such as the UK Biobank^76^, the European Genome-phenome Archive^77^ and the Cancer Imaging Archive^78^ have been created to help understand these factors, and contain large amounts of multiomics and imaging data. Furthermore, new technologies such as single-cell^79^ and spatial multiomics^80^ produce enormous quantities of data that need to be integrated and reduced to be understood. DLVPM is ideally suited for this task, as it is able to link arbitrarily many data modalities, including both structured and unstructured data. In this investigation, we showed how DLVPM can be used to construct a global model of breast cancer as an illness. Using this method, wholly different but connected manifestations of the same underlying illness can be understood with reference to the same neural network model.

## Methods

### DLVPM

Here, we give a general introduction and a full technical treatment of the DLVPM method. DLVPM can be thought of as a generalization of PLS-PM^23^. PLS-PM can be considered, in turn, to be a generalization of canonical correlation^81^. It is therefore natural to build an understanding of DLVPM with reference to these simpler methods. It is worth noting that we could have called our method Deep PLS-PM. However, we felt that DLVPM was more descriptive. We also wished to avoid confusion with the more popular PLS regression procedure.

The description of the DLVPM method we present here is broken into three basic parts:

1. a description of shallow (that is, non-deep-learning-based) methods for establishing correlations between different data types;
2. deep neural networks and notation;
3. a description of DLVPM, and how deep learning can be used to identify complex, nonlinear associations between different data types.

#### Canonical correlation analysis

Canonical correlation analysis (CCA) is a statistical method used to identify linear relationships between two or more sets of variables^81^. This method can be thought of as a generalization of linear least squares regression. The objective of CCA is to identify a relationship between two (or more) sets of variables, where there is no distinction between which variables are considered dependent and which are considered independent. This method identifies weights for each variable, such that the weighted sum of variables in each set is maximally correlated with the weighted sum of variables from the opposite set, assuming a linear relationship^81^.

Consider two matrices *X*_1_ and *X*_2_, where each row denotes one of *N* observations, and each column denotes *p*_1_ or *p*_2_ features for *X*_1_ and *X*_2_, respectively. CCA is optimized to find weight vectors *w*_1_ and *w*_2_ that maximize the association

$$\max_{{w}_{1},{w}_{2}}{\rm{corr}}({X}_{1}{w}_{1},{X}_{2}{w}_{2}).$$

We assume that the columns of *X*_1_ and *X*_2_ have been standardized to have a mean of zero and a standard deviation of one. Using the equation used to find Pearson’s correlation coefficient, we get

$$\max_{{w}_{1},{w}_{2}}\frac{{w}_{1}^{{\rm{T}}}{{X}_{1}}^{{\rm{T}}}{X}_{2}{w}_{2}}{{\Vert {X}_{1}{w}_{1}\Vert }_{2}{\Vert {X}_{2}{w}_{2}\Vert }_{2}}.$$

Notice that the denominator is simply a normalization term. Therefore, the canonical correlation objective can also be written as

$$\max_{{w}_{1},{w}_{2}}{w}_{1}^{{\rm{T}}}{{X}_{1}}^{{\rm{T}}}{X}_{2}{w}_{2}$$

subject to the constraints

$${\Vert{X}_{1}{w}_{1}\Vert}_{2}^{2}=1\,{\rm{and}}\,{\Vert{X}_{2}{w}_{2}\Vert}_{2}^{2}=1.$$

Here the vectors *X*_1_*w*_1_ and *X*_2_*w*_2_ are referred to as canonical variates.

In the original formulation, the canonical weights that maximize the association between the two data views are normally found using eigenvalue decomposition. It is possible to find multiple modes of variation using this method. Here the correlation between subsequent canonical variates is maximized subject to their being uncorrelated with other canonical variates. A total of *n*_dims_ = min(*p*_1_, *p*_2_) canonical variates can be extracted in this way. This can be written as

$$\max_{{W}_{1},{W}_{2}}{\rm{tr}}({({X}_{1}{W}_{1})}^{T}{X}_{2}{W}_{2})$$

subject to the orthogonalization constraints

$${({X}_{1}{W}_{1})}^{T}{X}_{1}{W}_{1}=I$$

and

$${({X}_{2}{W}_{2})}^{T}{X}_{2}{W}_{2}=I$$

where *W*_1_ and *W*_2_ are *p*_1_ × *n*_dims_ and *p*_2_ × *n*_dims_ matrices, respectively; and *I* is an *n*_dims_ × *n*_dims_ identity matrix.

#### Generalizing CCA

Hotelling’s original formulation of CCA was designed to identify associations between two data views. Researchers have generalized CCA to more than two data views^82^. There are a number of different ways in which this can be done. One way to generalize the CCA approach is to optimize the sum of correlations between different data views. This involves maximizing the following criteria:

$$\max_{{W}_{1},{W}_{2},\ldots, {W}_{K}}\mathop{\sum }\limits_{i,j,i\ne j}^{K}{\rm{tr}}({({X}_{i}{W}_{i})}^{{\rm{T}}}{X}_{j}{W}_{j})$$

where *K* is the total number of data views under analysis, and *i* and *j* index different data views, subject to the orthogonalization constraints

$${({X}_{i}{W}_{i})}^{{\rm{T}}}{X}_{i}{W}_{i}=I\;\forall i.$$

#### PLS-PM

The canonical correlation procedures described in the text above can be used to identify latent variables that are highly correlated between multiple data types. In some cases, we may wish to identify associations between some—but not all—data types. For example, a particular disease phenotype may have both genetic and environmental causes. It does not make much sense to try to link these genetic and environmental causes as they should be independent. Any model that attempts to link these data types may end up highlighting spurious effects.

The mathematical framework above, described with relation to generalized CCA, can be used to formulate a kind of structural-equation-modelling procedure called PLS-PM^83,84^. Using PLS-PM, it is possible to identify associations between prespecified data types. Utilizing this method, we specify which data types are connected with one another using a predefined adjacency matrix *C*. The adjacency matrix is a square matrix in which the elements *c*_*ij*_ represent connections between views *i* and *j*:

$$C=\left(\begin{array}{cc}\begin{array}{cc}{c}_{11} & {c}_{12}\\ {c}_{21} & {c}_{22}\end{array} & \begin{array}{cc}\cdots & {c}_{1K}\\ \cdot & {c}_{2K}\end{array}\\ \begin{array}{cc}\vdots & \cdot \\ {c}_{K1} & {c}_{K2}\end{array} & \begin{array}{cc}\cdot & \vdots \\ \cdots & {c}_{KK}\end{array}\end{array}\right)c_{ij}\in\{0,1\}\;\forall i,j$$

where *K* is again the total number of data types under analysis.

The optimization criteria can then be written as

$$\max_{{W}_{1},{W}_{2},\ldots, {W}_{K}}\mathop{\sum }\limits_{i,j,i\ne j}^{K}{c}_{ij}{\rm{tr}}\left({({X}_{i}{W}_{i})}^{{\rm{T}}}X_{j}W_{j}\right)$$

subject to the constraints

$${({X}_{i}{W}_{i})}^{{\rm{T}}}{X}_{i}{W}_{i}=I\;\forall i,$$

where *c*_*ij*_ represents the binary indexed elements of *C*.

Using PLS-PM, the full modelling process is normally referred to in two parts: the structural model and the measurement model. The structural model is the part of the model that defines which inter-relations are to be optimized between the data types; this information is stored in *C*. The measurement model is the part of each model (denoted by *X*_*i*_*W*_*i*_ ∀ *i*) that links individual features to latent variables in the path model^23^.

#### Deep neural networks and notation

Neural networks are computational models composed of layers of interconnected ‘neurons’ that perform calculations. During training, these networks adjust neuron connection weights via backpropagation, where they compute the gradient of a loss function (the difference between predicted and actual data) and iteratively update the network weights. The outputs of most neural networks can be written in the very general form:

$$\bar{Y}=F(X,U\;),$$

where $\overline{Y}$ is the network output and *F*(*X*, *U*) is some function that takes an input *X* and passes it though sets of weights and biases *U*. This could be many kinds of neural network, for example, a feed-forward neural network, a convolutional network or a transformer. The network output can be written more simply still as $\overline{Y}(X,U\;)$.

Each of the methods described in the text below relies on the last layer of the neural network having a linear projection weight. Treating this weight differently in the notation is crucial to understand the mechanisms by which DLVPM functions. Therefore, neural networks processing individual data types in DLVPM are written as $\overline{Y}(X,U,W\;)$, where *U* represents all weights and biases in the network up to the penultimate layer and *W* represents the weights on the last layer of the network. We use this very simple notation to denote neural networks in the rest of the text.

#### Deep canonical correlation

Andrew et al. developed a two-view form of CCA, which they termed deep CCA^85^. Deep CCA creates highly correlated representations of two data types by passing them through deep neural networks. The goal of the algorithm is to learn weights and biases of both data views such that we seek to maximize

$$\max_{{U}_{1},{U}_{2},{w}_{1}^{1},{w}_{1}^{2},\ldots, {w}_{1}^{{n}_{{\rm{dims}}}},{w}_{2}^{1},{w}_{2}^{2},\ldots, {w}_{2}^{{n}_{{\rm{dims}}}}}\mathop{\sum }\limits_{n=1}^{{n}_{{\rm{dims}}}}{\rm{corr}}\left(\;{\bar{y}}_{1}^{n}\left({X}_{1},{U}_{1},{w}_{1}^{n}\right),{\bar{y}}_{2}^{n}\left({X}_{2},{U}_{2},{w}_{2}^{n}\right)\right)$$

subject to the orthogonalization constraint

$${\left(\;{\overline{y}}_{1}^{\;n}\right)}^{\rm{T}}{\overline{y}}_{1}^{\;m}=1\,{\rm{when}}\,n=m\,{\rm{and}}\,{\left(\;{\overline{y}}_{1}^{\;n}\right)}^{\rm{T}}{\overline{y}}_{1}^{\;m}=0\,{\rm{when}}\,n\ne m,$$

where *n*_dims_ is the total number of canonical variates we wish to extract.

This optimization problem can be written in the matrix form as

$$ma{x}_{{U}_{1},{U}_{2},{W}_{1},{W}_{2}}tr({\overline{Y}}_{1}{({X}_{1},{U}_{1},{W}_{1})}^{\rm{T}}{{\overline{Y}}_{2}}({X}_{2},{U}_{2},{W}_{2}))$$

subject to the orthogonalization constraint

$${{\overline{Y}}_{1}}^{\rm{T}}{\overline{Y}}_{1}=I\,{\rm{and}}\,{{\overline{Y}}_{2}}^{\rm{T}}{\overline{Y}}_{2}=I$$

where ${\bar{Y}}_{i}$ is a column-wise concatenation of ${\bar{Y}}_{i}={\bar{y}}_{i}^{1}\leftrightarrow {\bar{y}}_{i}^{2}\leftrightarrow \ldots \leftrightarrow {\bar{y}}_{i}^{{n}_{\rm{dims}}}$ (↔ signifies the column-wise concatenation of CCA factors) and *I* is the identity matrix. Here *W*_1_ and *W*_2_ represent the set of all weights ${w}_{1}^{1},{w}_{1}^{2},\ldots ,{w}_{1}^{{n}_{\rm{dims}}}$ and ${w}_{2}^{1},{w}_{2}^{2},\ldots ,{w}_{2}^{{n}_{\rm{dims}}}$, respectively.

Andrew et al.’s formulation of this procedure operates by taking the derivative of the cross-covariance matrix between the data views. However, this approach is difficult to generalize to more than two data views. Wang et al.^86^ formulated an iterative least squares approach to this method. This involves minimizing the loss

$$L({X}_{1},{X}_{2},{U}_{1},{U}_{2},{W}_{1},{W}_{2})=\frac{1}{2}{\left\Vert \frac{{\bar{Y}}_{1}({X}_{1},{U}_{1},{W}_{1})}{{\bar{Y}}_{1}{\Vert ({X}_{1},{U}_{1},{W}_{1})\Vert }}-\frac{{\bar{Y}}_{2}({X}_{2},{U}_{2},{W}_{2})}{{\Vert {\bar{Y}}_{2}({X}_{2},{U}_{2},{W}_{2})\Vert }}\right\Vert}_{F}^{2}$$

subject to the orthogonalization constraint given above. We use a similar iterative least squares regression approach in the present investigation.

#### DLVPM

The goal of the DLVPM algorithm is to identify orthogonal modes of association between data views connected by the user-defined adjacency matrix *C*. As before, the adjacency matrix is a square matrix in which the elements *c*_*ij*_ represent connections between views *i* and *j*. This method is essentially a deep analogue of PLS path modelling. This adjacency matrix is often referred to as the structural or path model.

We therefore seek to maximize

$$ma{x}_{{W}_{1},{W}_{2},\ldots, {W}_{K},{U}_{1},{U}_{2},\ldots, {U}_{K}}\mathop{\sum }\limits_{i,j,i\ne j}^{K}{c}_{ij}tr({\bar{Y}}_{i}{({X}_{i},{U}_{i},{W}_{i})}^{T}{\bar{Y}}_{j}(X_{j},U_{j},W_{j}))$$

subject to the orthogonalization constraint

$${{\bar{Y}}_{i}}^{\rm{T}}{\bar{Y}}_{i}=I\;\forall i.$$

Taking the iterative regression approach followed by Wang et al. and described with reference to classical canonical correlation and PLS-PM earlier in the text, we can maximize the association between network outputs by minimizing the loss

$$\begin{array}{l}L({X}_{1},{X}_{2},\ldots, {X}_{K},{W}_{1},{W}_{2},\ldots, {W}_{K},{U}_{1},{U}_{2},\ldots, {U}_{K})\\=\mathop{\sum }\limits_{i,j,i\ne j}^{K}{c}_{ij}\dfrac{1}{2}{\left\Vert\displaystyle\frac{{\bar{Y}}_{i}({X}_{i},{U}_{i},{W}_{i})}{{\Vert {\bar{Y}}_{i}({X}_{i},{U}_{i},{W}_{i})\Vert }}-\displaystyle\frac{{\bar{Y}}_{j}(X_{j},U_{j},W_{j})}{{\Vert {\bar{Y}}_{j}(X_{j},U_{j},W_{j})\Vert }}\right\Vert}_{F}^{2}.\end{array}$$

#### Orthogonalization

The DLVPM algorithm can be split into two fundamental parts: an optimization step aimed at finding factors that are strongly correlated between data views, and a constraint that ensures DLVPM factors are orthogonal to one another. It is possible to identify a single factor of shared variance between sets without the orthogonalization step. The loss associated with finding a single DLVPM factor can be written as

$$\begin{array}{l}L({X}_{1},{X}_{2},\ldots, {X}_{K},{w}_{1},{w}_{2},\ldots, {w}_{K},{U}_{1},{U}_{2},\ldots, {U}_{K})\\=\mathop{\sum }\limits_{i,j,i\ne j}^{K}{c}_{ij}\,\displaystyle\frac{1}{2}{\left\Vert \displaystyle\frac{{\bar{y}}_{i}({X}_{i},{U}_{i},{w}_{i})}{{\Vert {\;\bar{y}}_{i}({X}_{i},{U}_{i},{w}_{i})\Vert }}-\displaystyle\frac{{\bar{y}}_{j}(X_{j},U_{j},w_{j})}{{\Vert {\;\bar{y}}_{j}(X_{j},U_{j},w_{j})\Vert }}\right\Vert}_{2}^{2}.\end{array}$$

In cases where we wish to identify more than a single factor of shared variance between data views, an orthogonalization step is required to decorrelate factors. We used two different approaches to orthogonalization in the present investigation. We first introduce an orthogonalization procedure inspired by classical PLS, which is used in the main part of this investigation. We also compared this approach to a whitening procedure, similar to the approach used in Wang et al.^86^.

#### Iterative orthogonalization

In the present investigation, we use a matrix deflation approach inspired by classical PLS-PM. This approach has the advantage that it maintains the proper ordering of DLVPM factors. During the forward pass through the network, data are orthogonalized with respect to previous DLVPM factors. Individual DLVPM factors are written as follows

$${\bar{y}}_{i}^{n}\left({X}_{i},{U}_{i},{w}_{i}^{n}\right).$$

The set of all DLVPM factors in a data view can be written as

$${\bar{Y}}_{i}({X}_{i},{U}_{i},{W}_{i}),$$

where ${\bar{Y}}_{i}$ is an *N* × *n*_*dims*_ matrix of DLVPM factors and *W*_*i*_ is a matrix of DLVPM weights. ${\bar{Y}}_{i}$ is a column-wise concatenation of ${\bar{Y}}_{i}={\bar{y}}_{i}^{1}\leftrightarrow {\bar{y}}_{i}^{2}\leftrightarrow {\ldots}\leftrightarrow {\bar{y}}_{i}^{{n}_{\rm{dims}}}$. Similarly, we define the matrix ${{\bar{Y}}_{i}}^{n}$ as the concatenation of all vectors from the first to the *n*th.

We denote the penultimate layer of the neural network with the notation *F*_*i*_(*X*_*i*_, *U*_*i*_). It is a well-known property of regression that the residual features, denoted by ${F}_{i}({X}_{i},{U}_{i}|{{\overline{Y}}_{i}}^{n})$, found in the regression

$${F}_{i}({X}_{i},{U}_{i}|{\bar{Y}}_{i}^{n})={F}_{i}({X}_{i},{U}_{i})-{\bar{Y}}_{i}^{n}{\left({\bar{Y}}_{i}^{\;{n}^{\rm{T}}}{\bar{Y}}_{i}^{\;n}\right)}^{-1}{\bar{Y}}_{i}^{{\;n}^{\rm{T}}}{F}_{i}\left({X}_{i},{U}_{i}\right),$$

are orthogonal to ${\bar{Y}}_{i}^{\;n}$ (utilizing the Moore–Penrose pseudo-inverse). We use this mechanism to identify orthogonal modes of variation using DLVPM.

We can then write the loss for the *n*th extracted latent factor, for the *i*th data view as

$${L}_{i}^{n}\left({X}_{i},\,{U}_{i},{w}_{i}^{n}\right)=\mathop{\sum }\limits_{j,\;j\ne i}^{K}{c}_{ij}\frac{1}{2}{\left\Vert {\;\bar{y}}_{j}^{n}(X_{j},U_{j})-\left({F}_{i}\left({X}_{i},{U}_{i}\right)\left|{\bar{Y}}_{i}^{n-1}\right){w}_{i}^{n}\right.\right\Vert }_{2}^{2}$$

given that $\,{L}_{i}^{n}$ is a sum of regression problems. We can then write the total loss for the *i*th data view as follows

$${L}_{i}({X}_{i},{U}_{i},{W}_{i})=\mathop{\sum }\limits_{n=1}^{{n}_{\rm{dims}}}{L}_{i}^{n}=\mathop{\sum }\limits_{n=1}^{{n}_{\rm{dims}}}\mathop{\sum }\limits_{j,\;j\ne i}^{K}{c}_{ij}\frac{1}{2}{\left\Vert {\bar{\;y}}_{j}^{n}\left(X_{j},U_{j}\right)-\left({F}_{i}({X}_{i},{U}_{i})|{\bar{Y}}_{i}^{n-1}\right){w}_{i}^{n}\right\Vert }_{2}^{2}$$

*L*_*i*_ is, therefore, written as a sum of the mean squared error losses across latent factors.

Similarly, the total loss can be calculated as the sum of losses across all data views

$$\begin{array}{rcl}L({X}_{1},{X}_{2}\ldots {X}_{K},{W}_{1},{W}_{2}\ldots {W}_{K},{U}_{1},{U}_{2}\ldots {U}_{K})=&&\sum\limits_{i}^{K}{L}_{i} \\=&& \sum\limits_{i}^{K}\sum\limits_{n=1}^{n_{dims}}\sum\limits_{j,\,j\ne i}^{K} c_{ij}\frac{1}{2}\|{\bar{y}}_{j}^{n}(X_{j},U_{j})\\&&-(F_{i}(X_{i},U_{i})|{\bar{Y}}_{i}^{\,n-1})w_{i}^{\,n}{\|}_{2}^{2}\end{array}$$

Owing to the orthogonalization process introduced in the text above, this formulation meets the following constraints

$${\left(\;{\bar{y}}_{i}^{n}\right)}^{\rm{T}}{\bar{y}}_{i}^{m}=1\,{\rm{when}}\,{n}={m},{\left(\;{\bar{y}}_{i}^{n}\right)}^{\rm{T}}{\bar{y}}_{i}^{m}={0}\,{\rm{when}}\,{n}{\ne}{m},{\rm{for}}\,{\rm{i}}={1},{2},{\ldots},{K}.$$

It is worth noting at this point that due to these constraints,

$${\bar{Y}}_{i}^{{n}^{\rm{T}}}{\bar{Y}}_{i}^{n}=I,$$

where *I* is the identity matrix. This means that the orthogonalization procedure

$${F}_{i}({X}_{i},{U}_{i}|{\bar{Y}}_{i}^{\,n})={F}_{i}({X}_{i},{U}_{i})-{\bar{Y}}_{i}^{\,n}{\left({\bar{Y}}_{i}^{\,{n}^{\rm{T}}}{\bar{Y}}_{i}^{\,n}\right)}^{-1}{\bar{Y}}_{i}^{\,{n}^{\rm{T}}}{F}_{i}({X}_{i},{U}_{i})$$

simplifies to

$${F}_{i}\left({X}_{i},{U}_{i}|{\bar{Y}}_{i}^{\,n}\right)={F}_{i}({X}_{i},{U}_{i})-{\bar{Y}}_{i}^{\,n}{\bar{Y}}_{i}^{{\,n}^{\rm{T}}}{F}_{i}({X}_{i},{U}_{i}).$$

DLVPM minimizes this loss in an iterative fashion by calculating the gradients associated with each data view and updating the weights of these data views.

So far, the analysis of the DLVPM algorithm has preceded assuming that training is carried out on the entire dataset simultaneously. However, neural networks are usually trained on subsets or batches of data whereas orthogonality is a property of the full dataset. Orthogonalization requires estimating a covariance matrix.

$${\Sigma }_{{Y}_{i}{F}_{i}}=\,{{\bar{Y}}_{i}}^{\rm{T}}{F}_{i}\left({X}_{i},\,{U}_{i}\right)$$

We calculate the covariance matrices above during model training, by making an initial estimate of the covariance matrices using the first batch and then updating this estimate using parameter re-estimation with momentum for each batch. The batch-level covariance matrices for the first batch are written as follows

$${\Sigma }_{{Y}_{i}{F}_{i}{\rm{b}}_{0}}={{\bar{Y}}_{{i}_{{\rm{b}}_{0}}}}^{\rm{T}}{F}_{i}({X}_{{i}_{{\rm{b}}_{0}}},\,{U}_{i})$$

The global covariance matrices for the first batch are then initially estimated as

$${\Sigma }_{{Y}_{i}{F}_{i}}=\frac{N}{{\rm{b}}_{0}}{\Sigma }_{{Y}_{i}{F}_{i}{\rm{b}}_{0}},$$

where *N* is the total number of samples and *b*_0_ is the size of the first batch. In subsequent batch updates, covariance matrices are calculated as

$${\Sigma }_{{Y}_{i}{F}_{i}}\leftarrow {{{\rho }}\times \Sigma }_{{Y}_{i}{F}_{i}}+\left(1-{{\rho }}\right)\frac{N}{{\rm{b}}_{{\rm{t}}}}\,{\Sigma }_{{Y}_{i}{F}_{i}{\rm{b}}_{{\rm{t}}}},$$

where *ρ* is the momentum of the update, *N* is the total number of samples under analysis and *b*_t_ is the size of the current batch and *ρ* is a hyperparameter that defines how quickly the covariance matrices are updated using the current batch. In the present investigation, we used a value of *ρ* = 0.95, which represents a trade-off between maintaining stable, smooth updates and allowing sufficient responsiveness to changes in newer data.

This algorithm allows us to learn global matrices for orthogonalization, which can then be used during inference. Nevertheless, we found that using these covariance matrices during training were ineffective at enforcing orthogonality. This is likely because using global covariance matrices does not enforce orthogonality at the batch-wise level. This means that gradient updates can also be non-orthogonal. However, if we use batch-wise orthogonalization, this condition is strongly enforced.

Consider the subloss for a particular data view, for a particular dimension of shared variance:

$${L}_{i}^{n}=\mathop{\sum }\limits_{j,\,j\ne i}^{K}{c}_{ij}\frac{1}{2}{\left\Vert\,{\bar{y}}_{j}^{\,n}\left(X_{j},U_{j}\right)-\left({F}_{i}\left({X}_{i},{U}_{i}\right)\left|{\bar{Y}}_{i}^{\,n-1}\right){w}_{i}^{n}\right.\right\Vert}_{2}^{2}.$$

Taking the gradient of ${L}_{i}^{n}$ with respect to the weight ${w}_{i}^{n}$ gives

$$\frac{\partial {L}_{i}^{n}}{\partial {w}_{i}^{n}}=-{\left({F}_{i}\left({X}_{i},{U}_{i}\right)\left|{\bar{Y}}_{i}^{\,n-1}\right.\right)}^{\rm{T}}\mathop{\sum }\limits_{j,\,j\ne i}^{K}{c}_{ij}\left(\,{\bar{y}}_{j}^{n}\left(X_{j},U_{j}\right)-\left({F}_{i}\left({X}_{i},{U}_{i}\right)\left|{\bar{Y}}_{i}^{\,n-1}\right){w}_{i}^{n}\right.\right).$$

Using batch-wise covariance matrices, we get

$$\bigl((F_{i}(X_{i},U_{i}) \mid \bar Y_{i}^{\,n-1})\,w_{i}^{\,n}\bigr)^{\!\top}\, (F_{i}(X_{i},U_{i}) \mid \bar Y_{i}^{\,m-1})\,w_{i}^{\,m} \;=\;0$$

and

$$\bar y_{j}^{\,n\!\top}\,\bar y_{j}^{\,m}=0.$$

Therefore, the gradients are orthogonal.

$${\left(\frac{{\partial L}_{i}^{n}}{\partial {w}_{i}^{n}}\right)}^{\rm{T}}\frac{{\partial L}_{i}^{m}}{\partial {w}_{i}^{m}}=0$$

For these reasons, the algorithm we used functions differently in training and testing. During model training, we implement orthogonalization using batch-wise covariance matrices. Global covariance matrices are used during testing. This different behaviour during training and testing, using batch-wise and global parameters, respectively, is similar in purpose and implementation to the batch normalization layer. The full algorithm specifying this method is shown in Fig. 1 and Extended Data Fig. 1. Pseudo-code illustrating how this algorithm works is shown below:

**DLVPM with iterative orthogonalization**

**Input**: data matrices ${{{X}}}_{{{i}}}\in {{\mathbb{R}}}^{{{{N}}\times {{p}}}_{{i}}}$ for *i* = 1, 2…*K*. Initialization of the weights *W*_*i*_, *U*_*i*_ for each data view, momentum *ρ* and learning rate *η*. Randomly choose a mini-batch and extract data for each data view as ${{{{X}}}_{{{i}}}}_{{{{b}}}_{0}}$.

**During training**

**For** *t* = 0, 1, 2,…,*T*, do:

   Forward propagate through the network:

   **For** *i* = 1, 2,…,*K*, do:      

$${\bar{Y}}_{i}={F}_{i}\left({X}_{{i}_{{\rm{b}}_{\rm{t}}}},\,{U}_{i},{W}_{i}\right)$$

$$\bar{Y}_{i}\;\leftarrow\;\dfrac{\bar{Y}_{i}}{\;\|\bar{Y}_{i}\dfrac{N}{b_{t}}\|}$$

   **For** *i* = 1, 2,…,*K*, do:

      Compute the batch mean and variance:      

$${\mu }_{{\rm{b}}_{\rm{t}}}=\frac{1}{{\rm{b}}_{\rm{t}}}\mathop{\sum }\limits_{n=1}^{{\rm{b}}_{\rm{t}}}{F}_{i}\left({X}_{{i}_{{\rm{b}}_{\rm{t}}}},\,{U}_{i}\right)\quad{{\sigma }_{{\rm{b}}_{\rm{t}}}}^{2}=\frac{1}{{\rm{b}}_{\rm{t}}}\mathop{\sum }\limits_{n=1}^{{\rm{b}}_{\rm{t}}}{\left({F}_{i}\left({X}_{{i}_{{\rm{b}}_{\rm{t}}}},{U}_{i}\right)-{\mu }_{{\rm{b}}_{\rm{t}}}\right)}^{2}$$

$${F}_{i}\left({X}_{{i}_{{\rm{b}}_{\rm{t}}}},\,{U}_{i}\right){\rm{\leftarrow }}\left({F}_{i}\left({X}_{{i}_{{\rm{b}}_{\rm{t}}}},\,{U}_{i}\right)-{\mu }_{{\rm{b}}_{\rm{t}}}\right)/{\sigma }_{{\rm{b}}_{\rm{t}}}$$

      **For** *n* = 1, 2…n_dims_, do:

         **If** *n* = 1:            

$$\frac{\partial {L}_{i}}{\partial {w}_{i}^{1}}=\frac{\partial }{\partial {w}_{i}^{1}}\mathop{\sum }\limits_{n=1}^{{n}_{\rm{dims}}}\mathop{\sum }\limits_{j,\,j\ne i}^{K}{c}_{ij}\frac{1}{2}{\left\Vert \bar{y}_{j}^{1}\!\left(X_{j}, U_{j}\right)-{F}_{i}({X}_{i},\,{U}_{i}){w}_{i}^{1}\right\Vert }_{2}^{2}$$

$$\,{\bar{y}}_{i}^{1}={F}_{i}\left({X}_{{i}_{{\rm{b}}_{\rm{t}}}},{U}_{i}\right){w}_{i}^{1}$$

$$\,{\bar{y}}_{i}^{n}={\bar{y}}_{i}^{1}$$

         **Else**            

$${\Sigma \,}_{{Y}_{i}{F}_{i}{\rm{b}}_{\rm{t}}}^{n}=\,{\left({\bar{Y}}_{{{ib}}_{\rm{t}}}^{\,n-1}\right)}^{\rm{T}}{F}_{i}\left({X}_{{i}_{{\rm{b}}_{\rm{t}}}},\,{U}_{i}\right)$$

$${F}_{i}({X}_{i},\,{U}_{i})|{{\bar{Y}}_{i}}^{n-1}={F}_{i}({X}_{i},\,{U}_{i})-\,{\bar{Y}}_{{{ib}}_{\rm{t}}}^{n-1}{\Sigma \,}_{{Y}_{i}{F}_{i}{\rm{b}}_{\rm{t}}}^{n}\,$$

$$\frac{\partial {L}_{i}}{\partial {w}_{i}^{n}}=\frac{\partial }{\partial {w}_{i}^{n}}\mathop{\sum }\limits_{n=1}^{{n}_{\rm{dims}}}\mathop{\sum }\limits_{j,\,j\ne i}^{K}{c}_{ij}\frac{1}{2}{\left\Vert\bar{y}_{j}^{n}\!\left(X_{j}, U_{j}\right)-\left({F}_{i}\left({X}_{i},{U}_{i}\right)\left|{{\bar{Y}}_{i}}^{n-1}\right){w}_{i}^{n}\right.\right\Vert }_{2}^{2}$$

$${{\bar{y}}_{i}^{n}}=\left({F}_{i}\left({X}_{i},\,{U}_{i}\right)\left|{{\bar{Y}}_{i}}^{n-1}\right){w}_{i}^{n}\right.$$

$${{\bar{Y}}_{i}}^{n}={{\bar{Y}}_{i}}^{n-1}\leftrightarrow {\bar{y}}_{i}^{n}$$

      **If** *t* = 0

         Define global variables, moving mean and moving variance:         

$${\sigma }^{2}={{\sigma }_{{\rm{b}}_{\rm{t}}}}^{2},\mu ={\mu }_{{\rm{b}}_{\rm{t}}}$$

         Covariance matrix (for orthogonalization):         

$${\Sigma }_{{Y}_{i}{F}_{i}}=\frac{N}{{\rm{b}}_{0}}\times {\Sigma }_{{Y}_{i}{F}_{i}{\rm{b}}_{\rm{t}}}$$

      **Else**

         For subsequent batches, update the batch moving mean and moving variance:         

$$\sigma ={{\rho }}\times \sigma +\left(1-{{\rho }}\right)\times {\sigma }_{{\rm{b}}_{\rm{t}}}$$

$$\mu ={{\rho }}\times \mu +\left(1-{{\rho }}\right)\times {\mu }_{{\rm{b}}_{\rm{t}}}$$

         Update the moving covariance matrices:         

$${\Sigma }_{{Y}_{i}{F}_{i}}\leftarrow {{{\rho }}\times \Sigma }_{{Y}_{i}{F}_{i}}+\left(1-{{\rho }}\right)\times \frac{N}{{\rm{b}}_{\rm{t}}}\,\times {\Sigma }_{{Y}_{i}{F}_{i}{\rm{b}}_{\rm{t}}}$$

         Update the weights:      

$${{{W}}}_{{{i}}}{\rm{\leftarrow }}{{{W}}}_{{{i}}}-{{\eta }}\frac{\partial {L}_{i}}{\partial {W}_{i}}$$

$${{{U}}}_{{{i}}}{\rm{\leftarrow }}{{{U}}}_{{{i}}}-{{\eta }}\frac{\partial {L}_{i}}{\partial {U}_{i}}$$

**During inference**

**For** *i* = 1, 2…*K*, do:

   Forward propagate through the network:   

$${F}_{i}\left({X}_{{i}_{{\rm{b}}_{\rm{t}}}},\,{U}_{i}\right)$$

$${F}_{i}\left({X}_{{i}_{{\rm{b}}_{\rm{t}}}},\,{U}_{i}\right){\rm{\leftarrow }}\left({F}_{i}\left({X}_{{i}_{{\rm{b}}_{\rm{t}}}},\,{U}_{i}\right)-\mu \right)/\sigma$$

   **If** *n* = 1:      

$${{\bar{y}}_{i}^{1}=F}_{i}\left({X}_{{i}_{{\rm{b}}_{\rm{t}}}},\,{U}_{i}\right){w}_{i}^{1}$$

$${{\bar{Y}}_{i}}^{n}={\bar{y}}_{i}^{1}$$

   **Else**      

$${{\bar{y}}_{i}^{n}}=\left({F}_{i}\left({X}_{i},\,{U}_{i}\right)\left|{{\bar{Y}}_{i}}^{n-1}\right)\right.{w}_{i}^{n}=\left({F}_{i}\left({X}_{i},\,{U}_{i}\right)-\,{\bar{Y}}_{{{ib}}_{\rm{t}}}^{\,n}{\Sigma \,}_{{Y}_{i}{F}_{i}}^{n}\right){w}_{i}^{n}$$

$${{\bar{Y}}_{i}}^{n}={{\bar{Y}}_{i}}^{n-1}\leftrightarrow {\bar{y}}_{i}^{n}$$

#### Whitening

Whitening offers a different way of orthogonalizing DLVs. This method of orthogonalization was used by Wang et al. in their formulation of deep CCA^86^.

Using the definitions of *Y*_*i*_ and *W*_*i*_ outlined earlier in the text, we can write the objective as

$$\begin{array}{l}L({X}_{1},{X}_{2},\ldots {X}_{k},{W}_{1},{W}_{2},\ldots {W}_{k},{U}_{1},{U}_{2}\ldots {U}_{K})=\\\mathop{\sum }\limits_{i,j,i\ne j}^{K}{c}_{ij}\displaystyle\frac{1}{2}{\left\Vert \frac{{F}_{i}({X}_{i},\,{U}_{i}){W}_{i}}{{\Vert {F}_{i}({X}_{i},{U}_{i}){W}_{i}\Vert }}-\,\frac{{F}_{j}(X_{j},\,U_{j})W_{j}}{{\Vert {F}_{j}(X_{j},U_{j})W_{j}\Vert }}\right\Vert}_{F}^{2}\end{array}$$

subject to

$${{\bar{Y}}_{i}}^{\rm{T}}{\bar{Y}}_{i}=I\;\forall i.$$

We note that if we multiply *Y*_*i*_ by the matrix square root of its inverse, we get the following.

$${\bar{Y}}_{i}\leftarrow {\left({{\bar{Y}}_{i}}^{\rm{T}}{\bar{Y}}_{i}\right)}^{-1/2}{\bar{Y}}_{i}$$

Then, the columns of *Y*_*i*_, representing different modes of variation, are uncorrelated. In other words, the orthogonality condition is met.

We introduce another algorithm, which again minimizes the global loss by iteratively minimizing the sum of the squared loss between each data view and connected data views. Using the whitening approach, we iteratively minimize the loss as follows

$$\begin{array}{l}L({X}_{1},{X}_{2},\ldots {X}_{k},{W}_{1},{W}_{2},\ldots {W}_{k},{U}_{1},{U}_{2}\ldots {U}_{K})\\=\mathop{\sum }\limits_{i,\;j,i\ne j}^{K}{c}_{ij}\frac{1}{2}{\left\Vert {\left({\bar{Y}_{j}}^{\rm{T}}\bar{Y}_{j}\right)}^{-1/2}\bar{Y}_{\!j}-{F}_{i}({X}_{i},{U}_{i}){W}_{i}\right\Vert }_{F}^{2}.\end{array}$$

As was the case when minimizing the loss using the iterative orthogonalization approach specified above, when trained at the batch-wise level, we must estimate a global covariance matrix. We do this in a similar manner to the way in which we estimated global covariance matrices using the iterative orthogonalization approach. As noted in the explanation of the iterative orthogonalization algorithm, training using deep learning is generally carried out at the batch-wise level.

For the first batch, in each data view, the covariance matrix is initially estimated as follows.

$${\Sigma }_{{Y}_{i}{Y}_{i}}=\bar{Y}_{{i}_{{\rm{b}}_{0}}}^{\rm{T}}\bar{Y}_{{i}_{{\rm{b}}_{0}}}$$

Subsequent batches are then estimated as follows.

$${\Sigma }_{{Y}_{i}{Y}_{i}}\leftarrow {{{\rho }}\times \Sigma }_{{Y}_{i}{Y}_{i}}+\,(1\,-{{\rho }})\frac{N}{{\rm{b}}_{\rm{t}}}\,{\Sigma }_{{Y}_{i}{Y}_{i}{\rm{b}}_{\rm{t}}}$$

The full pseudo-code for estimating a DLVPM model using the whitening orthogonalization approach is given below. Note that finding the matrix inverse can be computationally intensive for large embedding sizes. Using the Cholesky decomposition can be substantially quicker in finding ${({\bar{Y}_{j}}^{\rm{T}}\bar{Y}_{j})}^{-1/2}\bar{Y}_{j}$. Therefore, this is offered as an option in the DLVPM toolbox.

**DLVPM with whitening**

**Input:** data matrices ${{{X}}}_{{{i}}}\in {{\mathbb{R}}}^{{{{N}}\times {{p}}}_{{i}}}$ for *i* = 1, 2…*K*. Initialization of the weights *W*_*i*_, *U*_*i*_ for each data view, momentum *ρ* and learning rate *η*. Randomly choose a mini-batch and extract data for each data view as ${{X}_{i}}_{{\rm{b}}_{0}}$.

**During training**

**For** *t* = 0, 1, 2…*T*, do:

   Forward propagate through the network:

   **For** *i* = 1, 2…*K*, do:      

$$\bar{Y}_{i}={F}_{i}\left({X}_{{i}_{{\rm{b}}_{\rm{t}}}},\,{U}_{i},{W}_{i}\right)$$

$$\,\bar{Y}_{i}\,\leftarrow {\,{\Sigma }_{{Y}_{i}{Y}_{i}}}^{-1/2}\bar{Y}_{i} $$

   **For** *i* = 1, 2…*K*, do:

      Compute the batch mean and variance:      

$${\mu }_{{\rm{b}}_{\rm{t}}}=\frac{1}{{\rm{b}}_{\rm{t}}}\mathop{\sum }\limits_{n=1}^{{\rm{b}}_{\rm{t}}}{F}_{i}\left({X}_{{i}_{{\rm{b}}_{\rm{t}}}},\,{U}_{i}\right)\quad{{\sigma }_{{\rm{b}}_{\rm{t}}}}^{2}=\frac{1}{{\rm{b}}_{\rm{t}}}\mathop{\sum }\limits_{n=1}^{{\rm{b}}_{\rm{t}}}{\left({F}_{i}\left({X}_{{i}_{{\rm{b}}_{\rm{t}}}},{U}_{i}\right)-{\mu }_{{\rm{b}}_{\rm{t}}}\right)}^{2}$$

$${F}_{i}({X}_{{i}_{{\rm{b}}_{\rm{t}}}},\,{U}_{i}){\rm{\leftarrow }}({F}_{i}({X}_{{i}_{{\rm{b}}_{\rm{t}}}},\,{U}_{i})-{\mu }_{{\rm{b}}_{\rm{t}}})/{\sigma }_{{\rm{b}}_{\rm{t}}}$$

$$\frac{\partial {L}_{i}}{\partial {W}_{i}}=\frac{\partial }{\partial {W}_{i}}\mathop{\sum }\limits_{j,\,j\ne i}^{K}{c}_{ij}\frac{1}{2}{\left\Vert \bar{Y}_{j}-{F}_{i}({X}_{{i}_{{\rm{b}}_{\rm{t}}}},{U}_{i}){W}_{i}\right\Vert}_{F}^{2}$$

      **If** *t* = 0:

         Define global variables, moving mean and moving variance:         

$${\sigma }^{2}={{\sigma }_{{\rm{b}}_{\rm{t}}}}^{2},\mu ={\mu }_{{\rm{b}}_{\rm{t}}}$$

         Covariance matrices (for orthogonalization):         

$$\,{\Sigma }_{{Y}_{i}{Y}_{i}}=\frac{N}{{\rm{b}}_{0}}\times {\Sigma }_{{Y}_{i}{Y}_{i}{\rm{b}}_{\rm{t}}}$$

      **Else**

         For subsequent batches, update the batch moving mean and moving variance:         

$$\sigma ={{\rho }}\times \sigma +\left(1-{{\rho }}\right)\times {\sigma }_{{\rm{b}}_{\rm{t}}}$$

$$\mu ={{\rho }}\times \mu +\left(1-{{\rho }}\right)\times {\mu }_{{\rm{b}}_{\rm{t}}}$$

         Update the moving covariance matrices:         

$${\Sigma }_{{Y}_{i}{Y}_{i}}\leftarrow {{{\rho }}\times \Sigma }_{{Y}_{i}{Y}_{i}}+\,(1\,-{{\rho }})\times \frac{N}{{\rm{b}}_{\rm{t}}}\,\times {\Sigma }_{{Y}_{i}{Y}_{i}{\rm{b}}_{\rm{t}}}$$

         Update the weights:      

$${{{W}}}_{{{i}}}{\rm{\leftarrow }}{{{W}}}_{{{i}}}-{{\eta }}\frac{\partial {L}_{i}}{\partial {W}_{i}}$$

$${{{U}}}_{{{i}}}{\rm{\leftarrow }}{{{U}}}_{{{i}}}-{{\eta }}\frac{\partial {L}_{i}}{\partial {U}_{i}}$$

**During inference**

**For** *i* = 1, 2…*K*, do:

   Forward propagate through the network:   

$${F}_{i}{(X}_{{i}_{{\rm{b}}_{\rm{t}}}},\,{U}_{i})$$

$$\bar{Y}_{i}=(({F}_{i}({X}_{{i}_{{\rm{b}}_{\rm{t}}}},\,{U}_{i})-\mu )/\sigma){W}_{i}$$

### DLVPM-Twins

Although DLVPM is primarily designed to identify associations between multiple data views, it can also be used to find useful representations of a single data view, which can then be used for downstream tasks. When used in this manner, DLVPM falls into the class of methods called Siamese or twin networks. This class of methods has become popular across a wide range of fields in recent years. Twin architectures are trained by feeding a neural network with distorted versions of the same input. By using some kind of correlative loss on the output features (as is the case with DLVPM), the network is encouraged to learn representations that are invariant to these distortions, which can be useful in downstream analyses.

When using DLVPM in this way, the loss can be written as

$${\max }_{U,W}{\rm{tr}}\left({{\bar{Y}}_{A}({X}_{A},U,W\,)}^{\rm{T}}{\bar{Y}}_{B}({X}_{B},U,W\,)\right)$$

subject to the orthogonalization constraint

$${{\bar{Y}}_{A}}^{\rm{T}}{\bar{Y}}_{A}=I\,{\rm{and}}\,{{\bar{Y}}_{B}}^{\rm{T}}{\bar{Y}}_{B}=I.$$

Here *X*_*A*_ and *X*_*B*_ are augmented/distorted versions of the same input *X*. Note here that the weights and biases associated with the networks are the same for the entities we are seeking to maximize associations between. The network is then optimized to learn model outputs that are invariant to user-specified changes in the input.

DLVPM-Twins can be used with both iterative orthogonalization and whitening approaches. The algorithms defining these approaches are very similar to the full path-modelling algorithms. Therefore, they are not given here to avoid repetition.

### Removing confounds

Data are often subject to unwanted confounds that can affect the validity and generalizability of inferences made on these data. When assessing linear effects, these confounds can be removed by including them as covariates of no interest in a general linear model, or preregressing these unwanted effects from data before the analysis. We took a similar approach to removing confounding effects in neural networks. The last layer of a DLVPM model is linear. Therefore, removing confounding contributions before this layer will remove them entirely.

Here a set of confounds is denoted by an *N* × *D*_c_ matrix *C*, where *N* is the number of samples, and *D*_c_ is the number of confounds. *F*(*X*, *U*) has the same definition, given earlier in the text.

We implement the operation

$$(F(X,U\,)|C)=F(X,U\,)-C{({C}^{\rm{T}}C\,)}^{-1}{C}^{\rm{T}}F(X,U\,).$$

The matrix (*C*^T^*C*)^−1^*C*^T^ is known as the Moore–Penrose pseudo-inverse. It is a well-known result that columns of the resulting matrix (*F*(*X*, *U*)|*C*) are orthogonal to the columns of the matrix *C*.

When using DLVPM, we can use this approach to orthogonalize neuronal outputs with respect to a set of confounds in the penultimate layer. As projection layers are linear, the outputs of the measurement model will be orthogonal to these confounds. As with the DLV orthogonalization described earlier in the documentation, we must adapt this orthogonalization so that it is possible to train models using this approach at the batch-wise level.

The matrix

$$A={\left({C}^{\rm{T}}C\right)}^{-1}{C}^{\rm{T}}F\left(X,U\,\right)$$

can be split into two covariance matrices, namely,

$${{\Sigma }_{{{\rm{CC}}}}=C}^{\rm{T}}C$$

and

$${{\Sigma }_{{{\rm{CF}}}}=C}^{\rm{T}}F\left(X,U\,\right).$$

Batch-wise estimates of these matrices can be used to estimate full sample matrices. Batch-wise covariance matrices are written as

$${{\Sigma }_{\rm{CC}{\rm{b}}_{\rm{t}}}={C}_{{\rm{b}}_{\rm{t}}}}^{\rm{T}}{C}_{{\rm{b}}_{\rm{t}}}$$

and

$${{\Sigma }_{{CF}{\rm{b}}_{{\rm{t}}}}={C}_{{\rm{b}}_{{\rm{t}}}}}^{\rm{T}}F({X}_{{\rm{b}}_{{\rm{t}}}},\,U\,).$$

We can then carry out orthogonalization at the batch-wise level using

$$(F({X}_{{\rm{b}}_{\rm{t}}},U\,)|C)=F({X}_{{\rm{b}}_{\rm{t}}},U\,)-{C}_{{\rm{b}}_{\rm{t}}}{{{\sum }}_{CC{\rm{b}}_{\rm{t}}}}^{-1}{{\sum }}_{CF{\rm{b}}_{\rm{t}}}.$$

As in the case of carrying out orthogonalization between DLVs, we must also estimate full-sample covariance matrices so that we can carry out orthogonalization with respect to these parameters in unseen test data.

Global covariance matrices for the first batch are estimated as

$${\Sigma }_{\rm{CC}}=\frac{N}{{\rm{b}}_{0}}{{C}_{{\rm{b}}_{0}}}^{\rm{T}}{C}_{{\rm{b}}_{0}}$$

and

$${\Sigma }_{{CF}}=\frac{N}{{\rm{b}}_{0}}{{C}_{{\rm{b}}_{0}}}^{\rm{T}}F\left({X}_{{\rm{b}}_{0}},\,U\right).$$

In subsequent batches, these covariance matrices are updated with momentum:

$${\Sigma }_{\rm{CC}}\leftarrow {{{\rho }}\times \Sigma }_{\rm{CC}}+\left(1-{{\rho }}\right)\frac{N}{{\rm{b}}_{\rm{t}}}\,{\Sigma }_{\rm{CC}{\rm{b}}_{\rm{t}}}$$

and

$${\Sigma }_{{CF}}\leftarrow {{{\rho }}\times \Sigma }_{{CF}}+\left(1-{{\rho }}\right)\frac{N}{{\rm{b}}_{\rm{t}}}{\Sigma }_{{CF}{\rm{b}}_{\rm{t}}},$$

where *ρ* denotes the momentum.

At the model test time, these covariance matrices are then used to orthogonalize the signal that is forward propagated through the network, with respect to the confounding variables.

$$(F({X}_{{\rm{b}}_{\rm{t}}},U\,)|C)=F({X}_{{\rm{b}}_{\rm{t}}},U\,)-{C}_{{\rm{b}}_{\rm{t}}}{{{\sum }}_{CC}}^{-1}{{\sum }}_{CF}$$

Full pseudo-code illustrating this process is given below.

**Confound removal**

**Input**: Data matrices ${{{X}}}\in {{\mathbb{R}}}^{{{{N}}\times {{p}}}}$ and confound matrices $C\in {{\mathbb{R}}}^{{{N}}\times {{{D}}}_{{\rm{c}}}}$.

**During training**

**For** *t* = 0, 1, 2…*T*, do:

   Compute the batch mean and variance:

   $${\mu }_{{\rm{b}}_{\rm{t}}}=\frac{1}{{\rm{b}}_{\rm{t}}}\mathop{\sum }\limits_{n=1}^{{\rm{b}}_{\rm{t}}}{F}({X}_{{{\rm{b}}_{\rm{t}}}},{U})\quad{{\sigma }_{{\rm{b}}_{\rm{t}}}}^{2}=\frac{1}{{\rm{b}}_{\rm{t}}}\mathop{\sum }\limits_{n=1}^{{\rm{b}}_{\rm{t}}}{({F}({X}_{{{\rm{b}}_{\rm{t}}}},{U})-{\mu }_{{\rm{b}}_{\rm{t}}})}^{2}$$

   $$F({X}_{{\rm{b}}_{\rm{t}}},\,U\,){\rm{\leftarrow }}(F({X}_{{\rm{b}}_{\rm{t}}},\,U\,)\,-{\mu }_{{\rm{b}}_{\rm{t}}})/{\sigma }_{{\rm{b}}_{\rm{t}}}$$

   **If** *t* = 0:

      Define global variables, moving mean and moving variance:

      $${\sigma }^{2}={{\sigma }_{{\rm{b}}_{\rm{t}}}}^{2},\mu ={\mu }_{{\rm{b}}_{\rm{t}}}$$

      Covariance matrices (for orthogonalization):

      $${{\sum }}_{CC}=\frac{N}{{\rm{b}}_{0}}{{C}_{{\rm{b}}_{0}}}^{\rm{T}}{C}_{{\rm{b}}_{0}}$$ and

      $${{\sum }}_{CF}=\frac{N}{{\rm{b}}_{0}}{{C}_{{\rm{b}}_{0}}}^{\rm{T}}F({X}_{{\rm{b}}_{0}},U\,)$$

   **Else**

      For subsequent batches, update the batch moving mean and moving variance:

      $$\sigma ={\rho}\times \sigma +(1-{\rho})\times {\sigma }_{{\rm{b}}_{\rm{t}}}$$

      $$\mu ={\rho}\times \mu +(1-{\rho})\times {\mu }_{{\rm{b}}_{\rm{t}}}$$

      Update the moving covariance matrices:      

$${{\sum }}_{CC}\leftarrow {\rho}\times {{\sum }}_{CC}+(1-{\rho})\times \frac{N}{{\rm{b}}_{\rm{t}}}\times {{\sum }}_{CC{\rm{b}}_{\rm{t}}}$$

      and      

$${{\sum }}_{CF}\leftarrow {\rho}\times {{\sum }}_{CF}+(1-{\rho})\times \frac{N}{{\rm{b}}_{\rm{t}}}\times {{\sum }}_{CF{\rm{b}}_{\rm{t}}}$$

      Use these covariance matrices to remove the confounding effects:   

$$\,(F({X}_{{\rm{b}}_{\rm{t}}},U\,)|C\,)=F({X}_{{\rm{b}}_{\rm{t}}},U\,)-{C}_{{\rm{b}}_{\rm{t}}}\times {{{\sum }}_{CC{\rm{b}}_{\rm{t}}}}^{-1}{{\sum }}_{CF{\rm{b}}_{\rm{t}}}$$

**During inference**

   Forward propagate through the network:   

$$F({X}_{{\rm{b}}_{\rm{t}}},U\,)$$

$$F({X}_{{\rm{b}}_{\rm{t}}},U\,)\leftarrow(F({X}_{{\rm{b}}_{\rm{t}}},U\,)-\mu )/\sigma$$

$$F({X}_{{\rm{b}}_{\rm{t}}},U\,)\leftarrow F({X}_{{\rm{b}}_{\rm{t}}},U\,)-{C}_{{\rm{b}}_{\rm{t}}}\times {{{\sum }}_{CC}}^{-1}{{\sum }}_{CF}$$

### TCGA data

We initially applied DLVPM to data from the TCGA study (https://portal.gdc.cancer.gov/). Here we only used breast cancer patients with a full complement of data including histological images, RNA-seq, miRNA-seq, methylation and SNVs. To ensure we only used the highest-quality data, we subjected it to several selection steps before its use. We only used samples with a tumour purity above 60%. This threshold was chosen to minimize contamination from non-cancerous cells, thereby reducing background noise and increasing the precision of genetic and epigenetic profiling. By focusing on samples with higher tumour purity, we aimed to obtain clearer insights into tumour-specific molecular pathways and genetic alterations. The acquisition site can have a strong effect on both omics and imaging data^32^. We introduced a method to remove the effect of acquisition site (see the ‘Removing confounds’ section). However, when a small number of samples is associated with a covariate, it is not possible to disentangle biological effects and effects driven by this nuisance covariate. Therefore, we only used data from acquisition sites that contributed at least ten samples to the TCGA study. We only used female participants. 758 patient samples had a full set of SNV, methylation, miRNA-Seq, RNA-Seq and histological data available for the full path-modelling analysis.

Both DLVPM-Twins and full DLVPM path-modelling analysis require the sampling of subsections of each whole slide image (WSI) called image tiles. The first step in that process was to identify which parts of the overall image contain histological tissue. We did this by calculating Sobel’s image gradient across the whole slide. We then split the tissue into 224 × 224-pixel sections, subsequently referred to as image tiles. This tile size is the input size required by the EfficientNetB0 architecture^36^ used throughout this study. This process was used at ×5, ×10 and ×20 magnifications. Tiles in which the average Sobel’s image gradient was over 15 for over 50% of the image were considered to contain enough tissue to be used in further analyses.

Omics data are very high dimensional. First, we reduced the data dimensionality for each omics modality. In the case of methylation and RNA-seq data, we did this by finding the genes with the top 10% highest variance. miRNA-seq data have much lower dimensionality; therefore, we used the top 50% here. Omics data such as RNA-seq are often heavily skewed. We subjected all omics data to a rank-based inverse Gaussian transform to remove this data skew. TCGA data are typically of very high quality. However, a very small fraction of the methylation data (0.054%) was missing and coded as NaNs. As all omics data are centred on zero after *z* normalization, we simply replaced these values with zeros, representing the mean, after normalization. This is common practice in multimodal data integration analyses^87^.

### DLVPM-Twins model specification and training

DLVPM-Twins model training comprises two steps: a step to train a convolutional-neural-network-based model to learn meaningful features from individual histological image tiles extracted from WSIs, and a step to predict tumoural properties at the patient/WSI level. The dataset was randomly divided into training (80%) and testing (20%) subsets. This training/testing subset was used for both DLVPM-Twins model training and full DLVPM path modelling.

#### Feature training

We trained the DLVPM-Twins model using the EfficientNetB0 convolutional architecture, pretrained on ImageNet, on tiles extracted at ×20 magnification. Networks were trained using a feed-forward head on this convolutional base. This consisted of two dense layers with an output size of 512 followed by two further dense layers with an output of 4,096, all with rectified linear unit activations. Dropout layers with a dropout rate of 0.2 are used between all dense layers to prevent overfitting. The two larger (4,096) dense layers represent the embedding layer and are only used during training; these are discarded for testing and the output of the after the first 512-output-size dense layer is used as the representation layer. This network is inspired by the network head chosen in the original paper detailing the Barlow twins method^75^. The DLVPM-Iterative method becomes numerically unstable with larger output sizes. For this reason, we used an output size of 128 here, which was the largest size that was usable before the method became numerically unstable at a batch size of 256. For the DLVPM-Twins pretraining, we used a batch size of 256, and a learning rate of 1 × 10^−4^. These parameters were selected on the basis of previously published results^75^. The Adam optimizer was used in all the cases. This hyperparameter selection process was also applied to VicReg and Barlow twins methods. This training to produce the DLVPM-Twins image model was carried out for 100 epochs.

#### Data augmentation

Here a single image tile was extracted at random from the WSIs of each patient included in a training batch. Each tile was then subjected to various data augmentations; specifically, tiles were rotated by up to ±20°; translated horizontally and vertically by up to 20%; sheared by 20% to introduce geometric distortions; zoomed by up to 20% to vary scale; horizontally flipped to diversify the dataset further; brightness adjustments were made within a 70%–130% range to mimic different lighting conditions; tiles were randomly converted to a greyscale-like effect with a 50% probability to prepare the model for variations in stain quality. The ‘reflect’ filling mode was used for newly created pixels. DLVPM-Twins was then trained to maximize the associations between tiles subjected to these different data augmentations. This process was then repeated for subsequent batches, with tiles again extracted at random for each new batch.

#### Prediction at WSI/patient level

Following model training on individual tiles, the trained convolutional model architecture was applied to 100 tiles randomly extracted from WSIs for each patient at ×20 magnification. For each patient WSI, global mean average pooling was carried out over DLVs extracted over all the tiles. This results in a single set of DLVs for each subject. A single-layer classification head was then trained on these DLVs to predict the molecular and histological statuses, and the presence/absence of the *TP53* mutation. The same procedure was used to train the VicReg and Barlow twins models; for these methods, we used the optimal hyperparameter choices specified in the original publications for these methods^31,75^. For the single-layer classification head, we used a batch size of 32 and a learning rate of 0.001, which are standard parameters.

For each of the methods compared here, model training took 2 h on a single A100 GPU. Feature extraction before classification took a further 1 h on the same hardware, for each method.

In the initial experiments, our DLVPM-Twins model, trained on the histology data, was used to predict the histological and molecular statuses of TCGA tumours.

##### Histological subtypes

Breast cancer is primarily categorized into several histological subtypes: invasive ductal carcinoma (which originates from breast ducts) and invasive lobular carcinoma (arising from the lobules). Less common types include inflammatory breast cancer, known for its aggressive nature and inflammatory symptoms, and triple-negative breast cancer, which lacks hormonal receptors and is particularly challenging to treat. Information on the percentages of patients with these different histological subtypes of cancer is given in Supplementary Table 1^88^.

##### Molecular subtypes

The PAM50 molecular classification system categorizes breast cancer into five distinct subtypes based on gene expression: luminal A, luminal B, HER2 enriched, basal like and normal like. These subtypes inform prognosis and treatment decisions, with luminal A typically having the best outcome and basal like, the poorest owing to its aggressive nature and lack of hormone receptors. Information on the percentages of patients with these different molecular subtypes of cancer is given in Supplementary Table 2^89^.

We only used histological/molecular subtypes for prediction when there were at least 30 instances of that classification in the training set. This threshold ensures reliable predictions by avoiding overfitting and capturing meaningful patterns. It aligns with standard practices requiring sufficient sample sizes for stable model training and generalizability.

### Full DLVPM model specification and training

Full DLVPM requires that we specify a neural network for processing different data types included in an analysis. In path-modelling parlance, these different models are known as measurement models. The full DLVPM model encompassing all the measurement models is illustrated in Extended Data Fig. 3.

#### Histological measurement model

The histological imaging data were processed using a network that aggregates effects visible in the WSI data at different magnifications. To obtain effects from histology at different magnifications, we trained a DLVPM-Twins model at ×5, ×10 and ×20 magnifications. Here we used the whitening formulation of the method owing to its increased numeric stability. The DLVPM-Twins network layers after the convolutional base were assigned as trainable in the DLVPM path model. A trainable feed-forward neural network was then used to combine these multi-magnification effects. *L*_1_ and *L*_2_ weight regularization using standard regularization rates of *L*_1_ = 0.01 and *L*_2_ = 0.01 were used for all layers containing learnable weights, to prevent overfitting. A dropout layer^90^ using a standard dropout rate of 0.5 was applied before the confound removal layer for the same purpose.

#### Omics measurement model

Each of the omics models uses the same general neural network structure. The model utilizes an embedding layer that reduces the dimensionality of the input to the square root of the initial gene count, a heuristic inspired by natural language processing to efficiently capture the essence of gene expression patterns. Subsequent reshaping introduces a pseudo-sequence dimension, enabling the application of a self-attention layer, which facilitates the model’s focus on critical gene interactions. The attention output, merged with the original input through a residual connection, ensures the preservation of initial gene expression information and incorporating learned interaction effects. As with the histological neural network, regularization was again applied to all layers at rates of *L*_1_ = 0.01 and *L*_*2*_ = 0.01. Again, a dropout layer using a standard dropout rate of 0.5 was applied before the confound removal layer.

Both histological and omics measurement models end with a custom neural network layer that partials out the effect of confounds using the Moore–Penrose pseudo-inverse. This approach is detailed later, and is illustrated in Extended Data Fig. 3.

Once the individual measurement models are specified, the DLVPM method is used to construct DLVs from each different data type that are maximally associated with DLVs from other data types connected by the user-specified path model. DLVPM path modelling used the same overall train–test split as DLVPM-Twins. For hyperparameter optimization, the training data were further partitioned into 80% training and 20% validation sets through random splitting. Hyperparameter tuning involved multiple runs using batch sizes of 32, 64, 128 and 256. We implemented an exponential decay strategy for the learning rate, starting from initial values of 1 × 10^−2^, 1 × 10^−3^ and 1 × 10^−4^ and decaying to a value ten times lower. A grid search approach was utilized to determine the optimal batch size and initial learning rate. The hyperparameter combination yielding the highest evaluation metric (mean correlation between modalities connected by the path model) was then selected for further use. Following the selection of hyperparameters, the model was retrained on the entire initial training dataset (80%) using the selected hyperparameters. Each training run was carried out for 300 epochs. Here the histological DLVPM-Twins training step took 6 h on a single A100 GPU. Full DLVPM model training then took 35 min on the same hardware, including hyperparameter selection.

### Multimodal methods

We benchmarked the performance of the shallow path-modelling method, PLS-PM, against DLVPM in the task these methods are optimized to carry out: identifying associations between latent variables connected by a path model. We then compared the performance of DLVPM with several other multimodal data integration methods in the task of identifying multiomic loci associated with the model as a whole, and in identifying multiomic loci associated with the histology data. As with the full DLVPM path-modelling analysis, in the case of histological data, we concatenated the multi-magnification features extracted using DLVPM-Twins (see the ‘DLVPM-Twins model specification and training’ section), and used these as inputs to the model.

### Shallow PLS-PM

PLS-PM is closely related to DLVPM, and can be thought of as the classical equivalent of this method. PLS-PM is designed to construct sets of latent variables that are optimally correlated between data types connected by a path model. There are two major types of PLS-PM algorithm: mode A and mode B^23^. Mode A involves optimizing the association between different data types. This approach requires the calculation of the matrix inverse of within-modality covariance matrices. This is not possible when the number of examples in the data modality is smaller than the number of features. Mode B PLS-PM solves this issue by replacing within-modality covariance matrices with identity matrices. As the data in the present application have many more features than samples, we used mode B PLS-PM for comparison with DLVPM. When training the shallow PLS model, we used the same processed data as for DLVPM.

### MOFA+

MOFA+ generates factors derived from multiomics data by modelling each omics dataset as a linear combination of latent factors, with dataset-specific weight matrices capturing the contribution of each feature to the factors^39,40^. It uses a probabilistic model with a Gaussian likelihood for continuous data and alternatives for other data types (for example, Bernoulli for binary data), along with sparsity-inducing priors to ensure interpretable factorization. Optimization is performed via variational inference, enabling the efficient estimation of the factors and associated weights and handling missing data. This approach allows MOFA+ to disentangle shared and data-specific sources of variation across modalities. We used MOFA+ with standard parameters.

### Multimodal autoencoder

The deep multimodal autoencoder^41^ is designed for data integration across multiple modalities by learning a joint representation of multiple input data types. It extends the standard autoencoder structure to handle multimodal data, where the encoder maps inputs from multiple data types into a shared latent space, and the decoder reconstructs each modality from this latent representation. The key idea is to optimize the joint representation such that it captures the shared information across modalities as well as allows for modality-specific reconstructions. The model is trained using a combination of reconstruction loss for individual modalities and cross-modal reconstruction, ensuring that the learned latent space is meaningful even when some modalities are missing.

We used a multimodal autoencoder that integrates data from histology, RNA-seq, methylation, miRNA-seq and SNVs. In this work, each modality has a dedicated encoder with dense layers, rectified linear unit activations and batch normalization, producing a latent representation of size 128. Encoded representations are concatenated into a shared bottleneck layer of size 5 (the same number of DLVs extracted by DLVPM), capturing cross-modal relationships. Decoders, mirroring the encoders, reconstruct inputs from modality-specific representations. Training minimizes the mean squared error loss for each modality using the shared bottleneck as the target, ensuring compact, shared latent representations and retaining modality-specific features. As with the DLVPM, we ran this model for 300 epochs.

### Mediation effects

Statistical mediation analysis examines how an independent variable influences a dependent variable through a mediator. It involves assessing three key pathways: the effect of the independent variable on the mediation (path A), the effect of mediator on dependent variable (path B) and the direct effect of independent variable on the dependent variable (path C′). The total effect of the independent variable (path C) is decomposed into the direct effect (path C′) and the indirect effect (path A × path B). To test for significant mediation, the significance of the indirect effect (path A × path B) is evaluated using methods like the Sobel test or bootstrapping. Mediation helps to understand the underlying mechanism of how an independent variable affects a dependent variable through a mediator.

The effect of DLVs constructed from methylation, miRNA-seq and SNV data should act indirectly on histology, with RNA-seq acting as a mediator. We tested for mediation effects using the ‘statsmodels’ package. Our mediation model designated the DLVs derived from methylation, miRNA-seq and SNV data as independent variables, RNA-seq data as the mediator and histological outcomes as the dependent variable. To assess the significance of the mediation effect, statsmodels uses a bootstrapping approach. By using bootstrapping, statsmodels does not rely on the assumption of normality for the indirect effect, making it a robust method for mediation analysis. The results of the bootstrapped mediation analysis provided an estimate of the size and significance of the indirect effects of methylation, miRNA-Seq and SNV data on histology through RNA-seq data.

### Individually significant effects

DLVPM is a method for identifying global associations between different data types. We carried out additional analyses to localize effects to individual genetic loci. We ran these analyses to determine both overall significance of individual genetic loci within the path-modelling analysis and significance of their association with histological data. To assess the overall significance of individual genetic loci in the path-modelling analysis, we applied the following procedure. For each multiomic data type, we calculated the harmonic mean of Pearson’s correlation values between each omics locus and the DLVs connected to that data type via the DLVPM path model in the testing set. We chose the harmonic mean over the arithmetic mean because it is more sensitive to smaller values, which was crucial for identifying loci connected to all the modalities in the path model. Since the harmonic mean is always positive, we used the arithmetic mean to determine whether associations were positive or negative.

We then used permutation testing to ascribe significance to the mean of these associations for each multiomic data type. Using permutation testing, it is possible to correct for multiple comparisons by using the maximal statistic across all loci (here the largest mean correlation coefficient) as the statistic of interest in the permutation distribution^91^. This procedure controls the family-wise error in the strong sense.

We used the same procedure when determining the significance of associations between the histology data and individual genetic loci. The only difference in this analysis was that we only calculated associations between the individual genetic loci, and the histology DLVs, rather than taking the mean association as our statistic of interest.

### GSEA

We carried out a GSEA on the ranking of correlations between the gene expression scores, quantified by RNA-seq, and DLVs from connected datasets (Methods). Results from these analyses are shown in Supplementary Fig. 1. GSEA was carried out using the fgsea package in R. This analysis used the gene set ‘Hallmarks’, downloaded from https://www.gsea-msigdb.org/gsea/msigdb/human/collections.jsp. This analysis was conducted using the default exclusion criteria, in which pathways with fewer than 15, or more than 500, genes were omitted from the analysis. Significance was determined using an adjusted *P*-value threshold of less than 0.1. The normalized enrichment score was utilized to evaluate the effect sizes.

### CPTAC replication

We replicated the primary DLVPM model, originally trained on the TCGA data, using data from the CPTAC project. CPTAC, an initiative by the National Cancer Institute, integrates proteomics, genomics and transcriptomics to advance our understanding of cancer biology, identify biomarkers and drive precision medicine. The project provides publicly available multiomics datasets, including those for breast cancer. For this study, we utilized data from prospectively collected, non-TCGA samples^37^. These CPTAC samples included miRNA-seq, RNA-seq, SNV and histology data, though methylation data were not available. A total of 105 samples with all four data types were included in our analysis. Molecular data were obtained from https://kb.linkedomics.org/ (ref. ^38^) and histology data were sourced from https://www.cancerimagingarchive.net/ (ref. ^78^).

### Survival analysis

After training the DLVPM model on TCGA, we predicted clinical outcomes using DLVs as predictors in a Cox proportional hazards regression model. In breast cancer, the progression-free interval is the recommended clinical endpoint^92^. The Cox model enables the aggregation of effects across multiple DLVs, providing a comprehensive risk assessment. TCGA has the benefit of extensive omics and imaging characterization. Nevertheless, TCGA has the limitation of short follow-up times and incomplete records, making it less reliable for analysing outcomes requiring extended follow-up or detailed survival trends.

To address this limitation, survival analysis was replicated and extended using the METABRIC dataset^93,94^. METABRIC offers a large, well-characterized cohort with extensive genomic and transcriptomic data, complementing the TCGA dataset. Importantly, METABRIC features a longer follow-up time, crucial for capturing long-term survival outcomes and disease progression patterns. This extended follow-up enables a more robust estimation of hazard ratios and better differentiation between short- and long-term prognostic factors.

The TCGA model was trained using histology, RNA-seq, methylation, miRNA-seq and SNV data, enabling a rich, multimodal approach to outcome prediction. However, for METABRIC, only RNA-seq and SNV data were available. Despite this limitation, METABRIC’s extended follow-up and large cohort size provided a robust platform for validating and extending the survival analysis, demonstrating the flexibility of DLVPM in adapting to varying data modalities. We used *n* = 1,980 subjects from METABRIC, with all the subjects having clinical, RNA-seq and SNV data. We also compared the performance of DLVPM in predicting survival trends with that of several other multimodal data integration methods. METABRIC data were obtained from https://www.cbioportal.org/study/summary?id=brca_metabric. Analyses were carried out using the lifelines package.

### Histological visualization

Our DLVPM model undergoes training using tissue tiles and, on completion, we deploy it to analyse each tile individually. This enhances our understanding of the tumour segments that exhibit the most pronounced effects for specific DLVs. This allows us to pinpoint and assess the tumour subsections that have the greatest influence on each DLV.

### Single-cell analysis

Single-cell data were obtained from https://www.ncbi.nlm.nih.gov/geo/query/acc.cgi?acc=GSE176078. We applied the RNA-seq component of the full DLVPM model, trained on the TCGA dataset to data from the single-cell breast cancer encyclopaedia^59^. The single-cell breast cancer encyclopaedia is a collection of 100,064 single cells with transcriptomic data, taken from 26 primary tumours including 11 ER+, 5 HER2+ and 10 TNBC, representing the three major clinical subtypes. These data were preprocessed in the same manner as the TCGA RNA-seq data.

### Cell-line data

Cell-line data were obtained from https://depmap.org/portal/. Of the available breast cancer cell-line data, 61 RNA-seq samples, 67 SNV samples, 50 miRNA-seq samples and 45 CRISPR–Cas9 samples were available. All these omics data were used in the analyses presented here. Pairwise associations between omics data types and CRISPR–Cas9 data utilized all the intersecting samples.

Omics data from the depmap project were preprocessed for use in the same manner as data from the TCGA dataset. Although methylation data were collected as part of this project, these data are of a different type to those collected as part of the TCGA project. These data were, therefore, not used as part of the current investigation. In some cases, data were not available for particular genes/loci. These genes/loci were replaced by columns of zeros. The DLVPM model was robust to these changes as it was trained with a dropout layer, simulating this effect.

As noted earlier, we used a confound layer to remove the effects of nuisance covariates when training the DLVPM model on the TCGA data. When we applied the model to the CCLE data, this layer was removed from the model as these covariates are not relevant for the CCLE data. We also used batch-level statistics to ensure that the DLVs were orthogonal in this new dataset.

### Spatial transcriptomics

Spatial transcriptomic data were obtained from https://www.10xgenomics.com/. At the time of the analysis, four breast cancer samples were available using the Xenium platform. DLVPM was initially applied to the TCGA data to parse intertumoural heterogeneity. Because histology data are trained on sections of tissue called tiles, it is possible to deconvolve tile-wide effects back into the image space. This allows us to visualize histologic heterogeneity across individual tumours. Recently, a range of spatial transcriptomic methods have been developed with the aim of quantifying heterogeneity in gene expression across individual tumours.

The Xenium platform, from 10x Genomics, is an in situ hybridization-based spatial transcriptomic method^70^. This platform provides subcellular transcript resolution for genes known to be important in breast cancer. We sought to identify relations between the DLVPM models, and genes found to be essential to the functioning of cells scoring highly on these models. The DLVPM histological model has a tile-wise resolution of 224 × 224 pixels. We extracted tile-wise histological DLVs, and calculated the association between these DLVs and the total number of transcripts of genes of interest in the matching tile, normalized by the total number of transcripts.

We assessed the significance of associations between DLV 1, and the genes CCND1, GATA3 and ESR1. As there is a high degree of spatial autocorrelation in these data, an uncritical application of Pearson’s coefficient will lead to inflated significance levels and type-1 errors. For this reason, we used a method to assess statistical significance that fully accounts for spatial autocorrelation^95^ using the SpatialPack package in R.

### Reporting summary

Further information on research design is available in the Nature Portfolio Reporting Summary linked to this article.

## Supplementary information

Supplementary Information Supplementary Figs. 1 and 2, Tables 1–3 and legends for Tables 4 and 5.

Reporting Summary

Supplementary Table 4 Sheets containing the Pearson’s correlation values between each genetic locus and the DLV that modality is connected to via the path model. DLVs are labelled from DLV1 to DLV5. Each mean correlation value also has a family-wise error-corrected significance level attached.

Supplementary Table 5 Sheets containing the Pearson’s correlation values between each genetic locus and the histological DLVs. DLVs are labelled from DLV1 to DLV5. Each mean correlation value also has a family-wise error-corrected significance level attached.

## Data Availability

All data used in this study are publicly available. The TCGA data are available at https://portal.gdc.cancer.gov/. Data from the single-cell breast cancer encyclopaedia can be downloaded from https://www.ncbi.nlm.nih.gov/geo/query/acc.cgi?acc=GSE176078. Cancer dependency map data are available at https://depmap.org/portal/. Spatial transcriptomic breast cancer data derived from the Xenium platform can be downloaded from https://www.10xgenomics.com/. METABRIC data for the survival analysis are available at https://www.cbioportal.org/study/summary?id=brca_metabric. Molecular data for the CPTAC study were obtained from https://kb.linkedomics.org/ (ref. ^38^) and the histology data were sourced from https://www.cancerimagingarchive.net/ (ref. ^78^).

## Code availability

Code implementing the DLVPM method is available via GitHub at https://github.com/alexjamesing/Deep_LVPM and via Zenodo at 10.5281/zenodo.15245782 (ref. ^96^). All other scripts used for plotting and analysis are available from the corresponding author on request.
