# Supplementary material for: Integrating multimodal cancer data using deep latent variable path modelling
Source: Nat Mach Intell. 2025 Jul 22;7(7):1053–75. doi: 10.1038/s42256-025-01052-4 (PMC12283373; doi:10.1038/s42256-025-01052-4)
Supplement: Supplementary file 1 — Supplementary Figs. 1 and 2, Tables 1–3 and legends for Tables 4 and 5. [file 42256_2025_1052_MOESM1_ESM.pdf]

nature machine intelligence

Supplementary information https://doi.org/10.1038/s42256-025-01052-4

Integrating multimodal cancer data using deep latent variable path modelling

In the format provided by the authors and unedited

Supplementary Information

The supplementary information contains two Supplementary Figures with captions, Supplementary Tables 1-3 and table captions for Supplementary Tables 1-5.

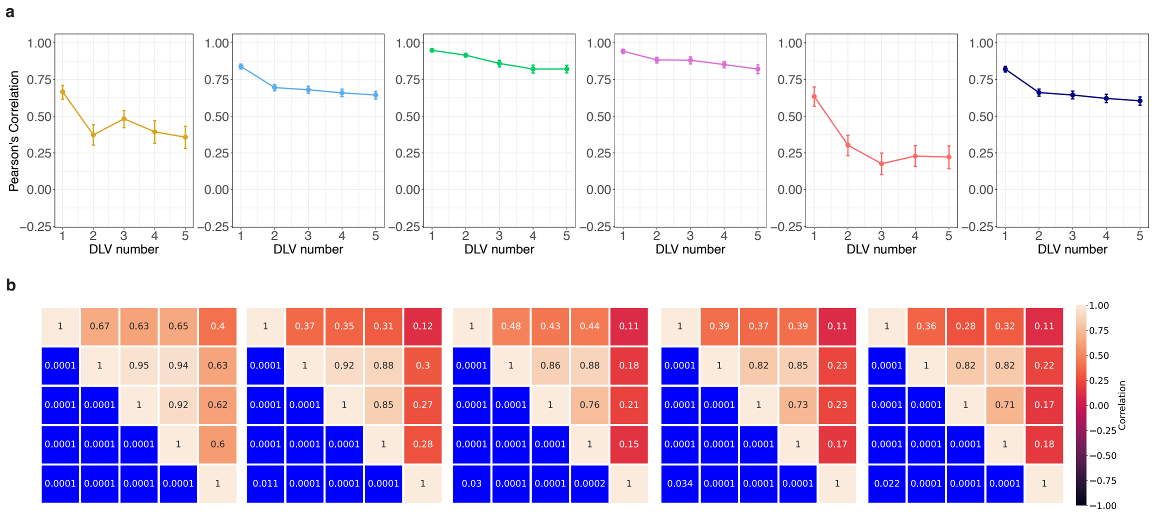

*Supplementary Figure 1: **Replication of main results using 5-fold cross-validation.** a: For each data type, these plots show the mean Pearson's correlation of each DLV, with DLVs from data types connected by the path model, in the TCGA dataset (n=758). The error bars on the plot denote mean-centred 95% bootstrapped confidence intervals. b: Association matrices for all five DLVs. Entries in the upper triangular part of the matrix indicate Pearson's correlation values between different data-types. Entries in the lower part of the matrix are significance values for these correlations, obtained using permutation testing.*

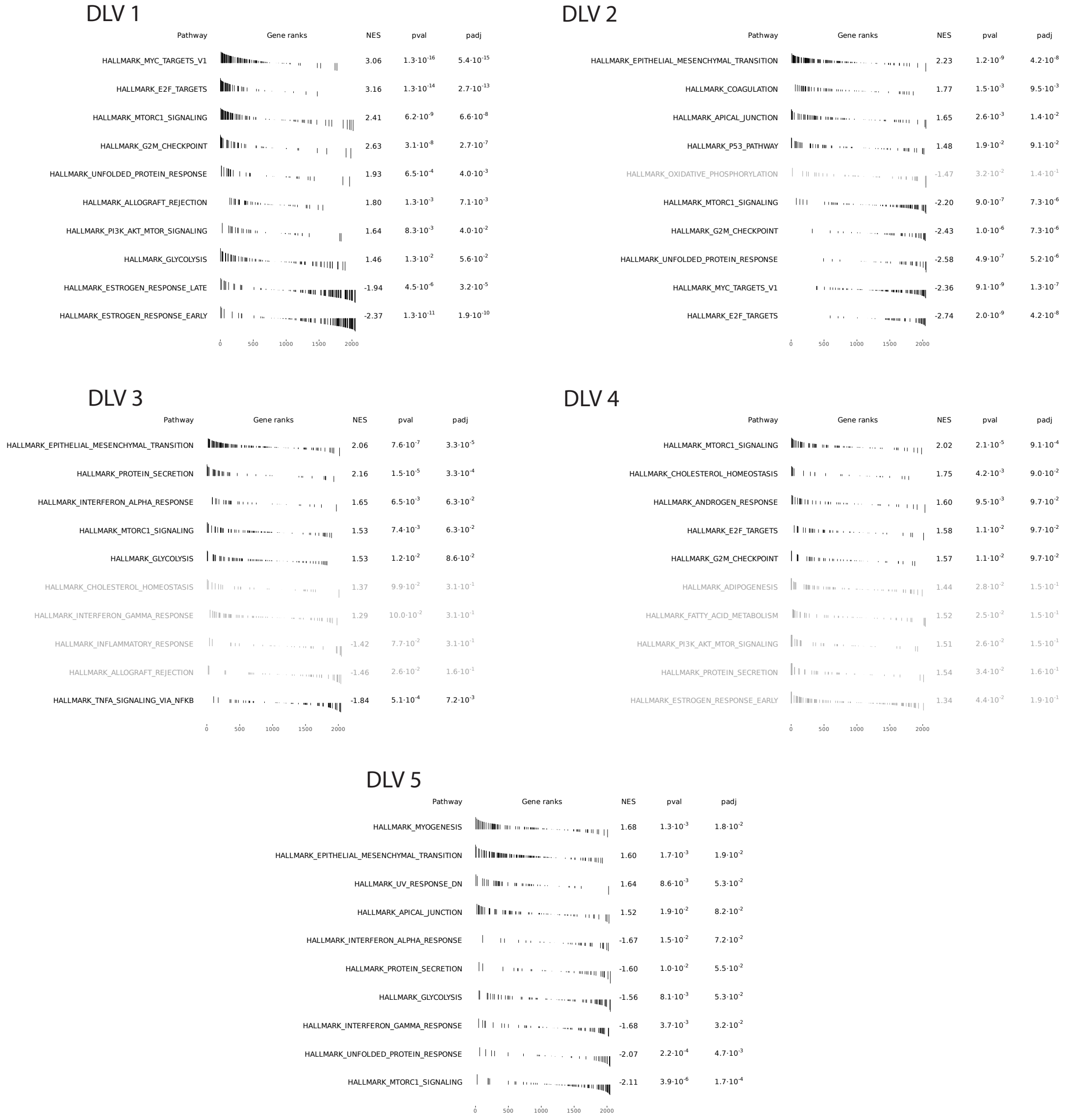

Supplementary Figure 2: ***This figure shows the top 10 most positively enriched, and top 10 most negatively enriched 'hallmarks of cancer' ontology terms for each of the DLVs extracted by DLVPM.*** *Terms that are non-significant at $p_{adj} > 0.1$ have been greyed out.*

Category	Count	Percentage
**NA**	2	0.26
**infiltratingductalcarcinoma**	577	76.12
**infiltratinglobularcarcinoma**	104	13.72
**medullarycarcinoma**	2	0.26
**metaplasticcarcinoma**	2	0.26
**mixedhistology(pleasespecify)**	22	2.90
**mucinouscarcinoma**	13	1.72
**others**	36	4.75

Supplementary Table 1: Distribution of patients across histological subtypes. The 'Category' column lists each histological subtype observed in the cohort, the 'Count' column provides the number of patients classified into each category, and the 'Percentage' column indicates the proportion of the total cohort that each subtype represents.

Category	Count	Percentage
**Basal**	94	12.40
**Her2**	46	6.06
**LumA**	323	42.61
**LumB**	151	19.92
**NA**	144	18.99

Supplementary Table 2: Distribution of patients across PAM50 molecular subtypes. The 'Category' column identifies each PAM50 subtype, the 'Count' column shows the number of patients classified within that subtype, and the 'Percentage' column reflects the fraction of the entire patient set corresponding to each molecular subtype.

	METABRIC Concordance Index	METABRIC Significance
**DLVPM-Iterative**	0.61	$p < 0.000001$
**DLVPM-Whiten**	0.61	$p < 0.000001$
**MOFA+**	0.61	$p < 0.000001$
**Autoencoder**	0.58	$p = 0.000122$
**PLS-PM**	0.60	$p < 0.000001$

Supplementary Table 3: Results of survival analysis comparing the performance of different in the METABRIC dataset. The models evaluated include DLVPM-Iterative, DLVPM-Whiten, MOFA+, Autoencoder, and PLS-PM. The concordance index and significance values are reported for both datasets, demonstrating the predictive power and statistical significance of each method in survival prediction.

Supplementary Table 4: Sheets from this table contain mean Pearson's Correlation values between each genetic loci, and DLVs that modality is connected to via the path model. DLVs are labelled from DLV1 to DLV5. Each of the mean correlation values also has a Family-wise error corrected (FWER) significance level attached.

Supplementary Table 5: Sheets from this table contain Pearson's Correlation values between each genetic loci, and the histological DLVs. DLVs are labelled from DLV1 to DLV5. Each of the mean correlation values also has a Family-wise error corrected (FWER) significance level attached.
