# Supplementary material for: Integrating multimodal cancer data using deep latent variable path modelling
Source: Nat Mach Intell. 2025 Jul 22;7(7):1053–75. doi: 10.1038/s42256-025-01052-4 (PMC12283373; doi:10.1038/s42256-025-01052-4)
Supplement: Supplementary file 2 — Reporting Summary [file 42256_2025_1052_MOESM2_ESM.pdf]

nature portfolio

Corresponding author(s): Jan Korbel
Last updated by author(s): Apr 25, 2025

Reporting Summary

Nature Portfolio wishes to improve the reproducibility of the work that we publish. This form provides structure for consistency and transparency in reporting. For further information on Nature Portfolio policies, see our Editorial Policies and the Editorial Policy Checklist.

Statistics

For all statistical analyses, confirm that the following items are present in the figure legend, table legend, main text, or Methods section.

n/a	Confirmed	
☐	☒	The exact sample size (*n*) for each experimental group/condition, given as a discrete number and unit of measurement
☒	☐	A statement on whether measurements were taken from distinct samples or whether the same sample was measured repeatedly
☐	☒	The statistical test(s) used AND whether they are one- or two-sided<br>*Only common tests should be described solely by name; describe more complex techniques in the Methods section.*
☐	☒	A description of all covariates tested
☐	☒	A description of any assumptions or corrections, such as tests of normality and adjustment for multiple comparisons
☐	☒	A full description of the statistical parameters including central tendency (e.g. means) or other basic estimates (e.g. regression coefficient) AND variation (e.g. standard deviation) or associated estimates of uncertainty (e.g. confidence intervals)
☐	☒	For null hypothesis testing, the test statistic (e.g. F, t, r) with confidence intervals, effect sizes, degrees of freedom and P value noted<br>*Give P values as exact values whenever suitable.*
☒	☐	For Bayesian analysis, information on the choice of priors and Markov chain Monte Carlo settings
☒	☐	For hierarchical and complex designs, identification of the appropriate level for tests and full reporting of outcomes
☐	☒	Estimates of effect sizes (e.g. Cohen's d, Pearson's r), indicating how they were calculated

Our web collection on statistics for biologists contains articles on many of the points above.

Software and code

Policy information about availability of computer code

Data collection	No data collection was performed.
Data analysis	Core analyses were carried out using Deep Latent Variable Path Modelling: https://github.com/alexjamesing/Deep_LVPM. Deep_LVPM was built using tensorflow 2.16.2. Plotting was carried out using ggplot2 (3.5.1) and Seaborn (0.13.0). Mediation analysis was carried out using statsmodels (0.14.4). Survival analyses were carried out using the lifelines package (0.30.0). Analyses on spatial transcriptomics data was carried out, in part, using the spatialpack package (0.4-1).

For manuscripts utilizing custom algorithms or software that are central to the research but not yet described in published literature, software must be made available to editors and reviewers. We strongly encourage code deposition in a community repository (e.g. GitHub). See the Nature Portfolio guidelines for submitting code & software for further information.

Data

Policy information about availability of data

All manuscripts must include a data availability statement. This statement should provide the following information, where applicable:

- Accession codes, unique identifiers, or web links for publicly available datasets
- A description of any restrictions on data availability
- For clinical datasets or third party data, please ensure that the statement adheres to our policy

All data used in this investigation is publicly available. TCGA data can be found at: https://portal.gdc.cancer.gov/. Data from the single cell breast cancer

encyclopaedia can be downloaded from: https://www.ncbi.nlm.nih.gov/geo/query/acc.cgi?acc=GSE176078. Cancer dependency map data can be found at: https://depmap.org/portal/. Spatial transcriptomic breast cancer data derived from the Xenium platform can be downloaded from: https://www.10xgenomics.com/. METABRIC data for the survival analysis can be found at: https://www.cbioportal.org/study/summary?id=brca_metabric. Molecular data for the CPTAC study were obtained from https://kb.linkedomics.org/, and histology data were sourced from https://www.cancerimagingarchive.net/.

Human research participants

Policy information about studies involving human research participants and Sex and Gender in Research.

Reporting on sex and gender	We only used female participants from the TCGA Breast Cancer study
Population characteristics	No stratifications were made on the basis of ethnicity/race.
Recruitment	No recruitment was carried out
Ethics oversight	Ethics oversight is not applicable as no recruitment was carried out

Note that full information on the approval of the study protocol must also be provided in the manuscript.

Field-specific reporting

Please select the one below that is the best fit for your research. If you are not sure, read the appropriate sections before making your selection.

☒ Life sciences ☐ Behavioural & social sciences ☐ Ecological, evolutionary & environmental sciences

For a reference copy of the document with all sections, see nature.com/documents/nr-reporting-summary-flat.pdf

Life sciences study design

All studies must disclose on these points even when the disclosure is negative.

Sample size	In each case, we used all available data that did not contradict our exclusion criteria. We did not make any statistical power calculations to determine sample size.
Data exclusions	For the TCGA data, we only used samples with a tumor purity over 60%. We also excluded data from aqusition sites that contributed less than 10 samples to the overall study. We only used female participants.
Replication	Results from the full TCGA path modelling analysis were replicated/extended on additional external datasets:<br>CPTAC: Replication of core multi-omic and histological associations, identified on a DLVPM model trained on TCGA data.<br>CCLE: Replication of core multi-omic associations identified on a DLVPM model trained on TCGA data.
Randomization	Data from the TCGA study was randomly allocated to training and testing datasets in a 80% to 20% split for both the DLVPM-Twins analysis, and the full DLVPM path modelling analysis.
Blinding	Does not apply here as group assignments are not made.

Reporting for specific materials, systems and methods

We require information from authors about some types of materials, experimental systems and methods used in many studies. Here, indicate whether each material, system or method listed is relevant to your study. If you are not sure if a list item applies to your research, read the appropriate section before selecting a response.

Materials & experimental systems

n/a	Involved in the study	
☒	☐	Antibodies
☒	☐	Eukaryotic cell lines
☒	☐	Palaeontology and archaeology
☒	☐	Animals and other organisms
☒	☐	Clinical data
☒	☐	Dual use research of concern

Methods

n/a	Involved in the study	
☒	☐	ChIP-seq
☒	☐	Flow cytometry
☒	☐	MRI-based neuroimaging
